# Association of Remnant Cholesterol Inflammatory Index with Stroke, Heart Disease and All-Cause Mortality Across Cardiovascular–Kidney–Metabolic Syndrome Stages 0–3: A National Cohort Study

**DOI:** 10.3390/nu18020205

**Published:** 2026-01-08

**Authors:** Huan Chen, Jing-Yun Wu, Hao Yan, Jian Gao, Chuan Li, Jia-Hao Xie, Xiao-Lin Wang, Ji-Long Huang, Dan Liu, Zhi-Hao Li, Chen Mao

**Affiliations:** 1 Department of Epidemiology, School of Public Health, Southern Medical University, Guangzhou 510515, China; chenhuanoool@163.com (H.C.); wu13622625756@126.com (J.-Y.W.); yanhao_0307@163.com (H.Y.); gaojian_2022@smu.edu.cn (J.G.); lichuan202105@163.com (C.L.); xiejiahao77777@163.com (J.-H.X.); 13728058502@163.com (X.-L.W.); 16620483557@163.com (J.-L.H.); liudan0717@smu.edu.cn (D.L.); zhihaoli2013@smu.edu.cn (Z.-H.L.); 2 National Institute of Health Data Science, Southern Medical University, Guangzhou 510515, China

**Keywords:** remnant cholesterol inflammatory index, stroke, all-cause mortality, CHARLS

## Abstract

**Background:** The Remnant Cholesterol Inflammatory index (RCII) has been proposed as a marker of insulin resistance and systemic inflammation. However, its associations with incident stroke, incident heart disease, and all-cause mortality among individuals with cardiovascular–kidney–metabolic (CKM) syndrome stages 0–3 remain uncertain. **Methods:** This longitudinal cohort study used data from the China Health and Retirement Longitudinal Study (CHARLS). The remnant cholesterol inflammatory index (RCII) was calculated as [RC (mg/dL) × hs-CRP (mg/L)]/10. Outcomes included incident stroke, incident heart disease, and all-cause mortality. Covariates were prespecified based on established risk factors. Cox proportional hazards models and restricted cubic spline (RCS) analyses were used to evaluate associations between RCII and each outcome. Long-term RCII patterns were identified using k-means clustering. Robustness was assessed using subgroup and sensitivity analyses. **Results:** The final study involved 6994 participants in the stroke and heart disease cohort and 7245 participants in the all-cause mortality cohort, all within CKM syndrome stages 0–3. Higher baseline RCII was associated with increased risks of stroke (HR = 1.55, 95% CI: 1.14–2.12) and all-cause mortality (HR = 1.67, 95% CI: 1.37–2.04) compared with the lowest quantile. Cumulative RCII showed a stronger association with all-cause mortality (HR for Q3 = 2.18, 95% CI: 1.54–3.11). RCS analysis suggested a J-shaped, non-linear association between cumulative RCII and all-cause mortality. (*p* for non-linearity < 0.05). K-means clustering further indicated that, relative to the reference group, cluster 2 (high-to-higher) had the highest risk of incident heart disease, whereas cluster 3 (high-to-moderate) had the highest risk of all-cause mortality. **Conclusions:** Higher RCII levels were associated with higher risks of stroke, heart disease, and all-cause mortality among individuals with CKM stages 0–3. RCII may serve as a promising biomarker for early risk stratification in clinic and prevention efforts in this population.

## 1. Introduction

Stroke is a leading cause of mortality and long-term disabilities globally, posing a significant public health challenge. It is estimated that approximately 7.5 million people died from stroke globally in 2021 [1,2], while coronary heart disease (CHD) caused 371,506 deaths in 2022 [3]. This trend highlights the urgency of reducing all-cause mortality and strengthening the prevention and control of cardiovascular health.

Extensive research has revealed a close, bidirectional relationship among stroke, chronic kidney disease (CKD), and metabolic disorders [4,5,6]. In its October 2023 presidential advisory, the American Heart Association (AHA) defined the cardiovascular-kidney-metabolic (CKM) syndrome as a systemic condition driven by the interaction of these three components [7]. Because CVD often coexists with hypertension, renal dysfunction, and metabolic abnormalities—sharing common pathophysiological pathways—the AHA emphasized the importance of early risk prediction and primary prevention in individuals at CKM stages 0–3 [7].

Atherosclerosis is the core pathological basis of stroke and heart disease, primarily driven by dyslipidemia and vascular inflammation [8,9]. Increasing evidence indicates that inflammation and metabolic dysregulation, particularly insulin resistance, jointly contribute to the residual stroke risk [10,11]. Remnant cholesterol (RC) refers to the cholesterol content of triglyceride-rich lipoproteins, including very-low-density lipoproteins (VLDL), intermediate-density lipoproteins (IDL), and chylomicron remnants. These particles can infiltrate the arterial wall, promote foam cell formation, and contribute to atherothrombosis [12]. Genetic and clinical evidence support a causal role of remnant cholesterol (RC) in myocardial infarction and ischemic stroke [13,14,15,16]. High-sensitivity C-reactive protein (hs-CRP) is a well-established biomarker of systemic inflammation [17,18,19,20]. Recent studies have indicated that hs-CRP may contribute to the residual risk of stroke [21]. CKM stages 0–3 represent an early risk spectrum in which metabolic dysregulation and low-grade inflammation may accelerate vascular injury [22]. Given the interplay between RC–related metabolic pathways and hs-CRP–related inflammatory pathways, a single biomarker may not fully capture stroke risk. Accordingly, combining these measures into the Remnant Cholesterol Inflammatory index (RCII) may improve risk prediction and reclassification for stroke and all-cause mortality [23]. An increasing number of studies have linked higher RCII to poorer stroke outcomes [24], and to a higher risk of cardiovascular disease among adults with CKM stages 0–3 [25]. Despite emerging evidence, data specifically among individuals with CKM stages 0–3 remain limited. Prior studies have often relied on a single baseline RCII and have rarely examined incident stroke, incident heart disease, and all-cause mortality jointly, or evaluated cumulative RCII and longitudinal patterns over time.

This study aimed to examine associations between RCII levels and the risks of stroke, heart disease, and all-cause mortality among individuals with CKM syndrome stages 0–3, to inform efforts to reduce stroke risk and overall disease burden.

## 2. Materials and Methods

### 2.1. Study Design and Population

This study used data from the China Health and Retirement Longitudinal Study (CHARLS), a nationally representative cohort of Chinese adults aged ≥45 years that collects detailed health and sociodemographic information. The study design and procedures have been described in detail elsewhere [26]. The study protocol complied with the Declaration of Helsinki and was approved by the Institutional Review Board (IRB) of Peking University. Written informed consent was obtained from all participants prior to the baseline survey.

**Figure 1:**
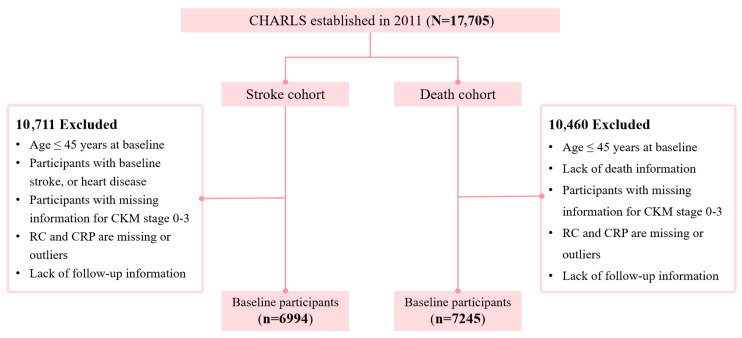
Flow chart of study subjects.

Data were drawn from multiple waves of CHARLS, from the baseline survey in 2011 (Wave 1) through the most recent follow-up in 2020 (Wave 5). Among the 17,708 participants initially enrolled (Figure 1), we excluded those with (1) missing baseline data on key exposure variables (hs-CRP, total cholesterol [TC], high-density lipoprotein cholesterol [HDL-C], or low-density lipoprotein cholesterol [LDL-C]); (2) a history of stroke, heart disease (including self-reported physician diagnosis), or CKM at baseline; (3) missing data on covariates required for model adjustment; or (4) loss to follow-up. After these exclusions, 6994 participants remained for the final analysis.

### 2.2. Definition of RCII

RCII, an integrative biomarker reflecting both inflammation and dyslipidemia, was calculated as: RCII = [RC (mg/dL) × hs-CRP (mg/L)]/10 [21]. Cumulative RCII (cuRCII) was calculated as follows: cuRCII = (RCII2011 + RCII2015)/2 × 3. This linear model integrates the measurements from Wave 1 (2011) and Wave 3 (2015) with a 3-year interval, based on prior RC and hs-CRP stability data.

### 2.3. Definition of CKM Syndrome Stages 0–3

According to the 2023 AHA advisory, CKM syndrome is classified into stages 0–4, reflecting progressive risk across metabolic, renal, and cardiovascular systems [7]. Stage 0 includes individuals with no CKM risk factors and normal metabolic and kidney function. Stage 1 involves obesity or impaired glucose metabolism. Stage 2 includes metabolic risk factors combined with moderate-to-high chronic kidney disease. Stage 3 denotes subclinical CVD or advanced CKD (more details see Supplementary Method S1).

### 2.4. Study Outcomes

The primary outcomes were the incidence of stroke, heart disease and all-cause mortality. Outcome ascertainment was primarily based on physician diagnoses self-reported by participants or reported by their immediate family members during the CHARLS follow-up interviews. The interviews included a standardized question: “Has a doctor ever told you that you have heart disease (including myocardial infarction, coronary heart disease, angina, congestive heart failure, or other heart problems) or stroke?” For participants who died during follow-up, the date and primary cause of death were determined from information provided by their relatives. Person-time for each participant was calculated from the baseline interview date until the first occurrence of a study outcome, death, the date of the last known follow-up, or the end of the study period (31 December 2020), whichever came first.

### 2.5. Covariates

Potential confounders were assessed at baseline using standardized questionnaires, physical examinations, and laboratory tests. Covariates were grouped as follows: (1) sociodemographic characteristics: age, sex, occupation (farmer vs. non-farmer), education level (illiterate vs. non-illiterate), and marital status (married vs. other); (2) lifestyle factors: smoking status (non-smoker vs. smoker), drinking status (non-drinker vs. drinker), and sleep duration; and (3) clinical and biochemical parameters: body mass index (BMI; kg/m^2^), systolic and diastolic blood pressure, dyslipidemia treatment, and a history of cancer or hypertension [24].

### 2.6. Statistical Analysis

Participants were classified into three RCII groups based on quartiles (Q1–Q3), and the cut points were not sex-specific. Normality of continuous variables was assessed using the Shapiro–Wilk test. Normally distributed continuous variables are presented as mean ± SD and compared using analysis of variance (ANOVA), whereas non-normally distributed variables are presented as median (IQR) and compared using the Kruskal–Wallis test. Categorical variables are reported as n (%) and compared using the chi-square test.

Cox proportional hazards models were used to examine associations of baseline RCII with CVD risk and all-cause mortality, and Kaplan–Meier curves were used for visualization. Outliers were excluded. Schoenfeld residual tests indicated no evidence of violations of the proportional hazard assumption in the adjusted models. For cumulative RCII, Cox regression with a four-knot restricted cubic spline (RCS) was used to assess potential non-linear dose–response relationships. K-means clustering was used to classify participants into four phenotypic clusters based on RCII, hs-CRP, and RC measured in Wave 1 (2011) and Wave 3 (2015). The number of clusters was determined using the elbow method (Supplementary Method S2). Cox models were then used to assess associations between cluster membership and the outcomes.

Subgroup analyses were performed, and potential effect modification was evaluated across age, sex, marital status, educational attainment, drinking status, smoking status, sleep duration, and CKM stage. Sensitivity analyses were performed to assess robustness by (1) excluding participants who experienced an outcome event within the first 2 years of follow-up to reduce potential reverse causation; (2) using multiple imputation for missing covariate data; and (3) re-estimating associations using alternative categorizations of the exposure (dichotomous, quartiles, and quintiles).

All statistical analyses were performed using R Studio (version 4.2.3). A two-sided *p* value < 0.05 was considered statistically significant for all analyses.

## 3. Results

### 3.1. Population Characteristics

This study included 6994 participants for stroke and heart disease analyses and 7245 participants for all-cause mortality analyses within CKM syndrome stages 0–3, among whom 52.3% were women, and the mean age was 59.15 ± 9.32 years. Over a median follow-up of 9 years, 653 deaths occurred. According to CKM staging, 669 participants were classified as stage 0, 1076 as stage 1, 4663 as stage 2, and 586 as stage 3.

As shown in Table 1, baseline characteristics were compared between participants who developed stroke and those who did not. Participants who developed stroke were older and had higher systolic and diastolic blood pressure and higher RC levels (all *p* < 0.001). The prevalence of hypertension and the proportion of CKM stage ≥ 2 were also greater among participants with higher RCII levels.

### 3.2. Associations of RCII with the Risk of Stroke, Heart Disease and All-Cause Mortality

Kaplan–Meier curves showed that higher RCII levels were associated with higher risks of incident stroke, incident heart disease, and all-cause mortality. The log-rank test indicated differences across RCII quartiles (*p* < 0.001), with a graded increase in risk across outcomes (Figure S1). Similar patterns were observed for RC and hs-CRP (Figures S2 and S3).

**Figure 2:**
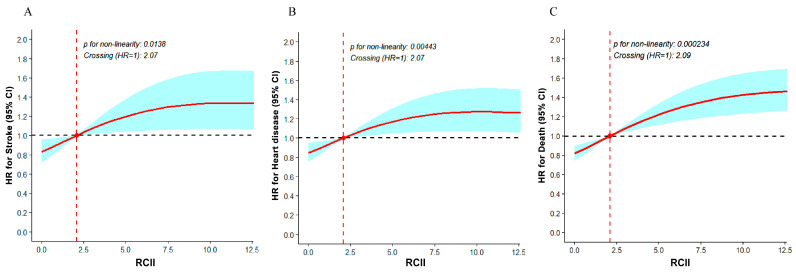
The association of RCII with the risk of stroke (**A**), heart disease (**B**) and all-cause mortality (**C**) with CKM syndrome stages 0–3. Adjusted age, sex, education level, occupation, marital Status, sleep duration, smoke status, drink status, SBP, DBP, BMI, cancer, hypertension, dyslipidemia treatment. Abbreviations: RCII, remnant cholesterol inflammatory index; HR, hazard ratio; CI, confidence interval.

RCS analyses indicated non-linear associations between RCII and stroke, heart disease, and all-cause mortality (all *p* for non-linearity < 0.05), with hazard ratios reaching a nadir at RCII levels of approximately 2.07, 2.07, and 2.09, respectively (Figure 2). Corresponding RCS analyses for RC and hs-CRP are shown in Figures S4 and S5.

In multivariable-adjusted Cox models (Table 2), stroke risk increased across RCII quartiles (*p* for trend < 0.05). The HR for Q3 was 1.55 (95% CI 1.14–2.12), and the population attributable fraction (PAF) was 14.0% (95% CI 4.3–22.2%). Participants in Q3 had a 40% higher risk of heart disease than those in Q1. The PAF of RCII for heart disease was 10.7% (95% CI: 3.2–17.4%). For all-cause mortality, the HR for Q3 was 1.67 (95% CI 1.37–2.04) and the PAF was 16.6% (95% CI 10.5–22.0%), with an upward trend across quartiles (*p* for trend < 0.05). Similar patterns were observed for RC and hs-CRP (Table S4).

### 3.3. Associations of Cumulative RCII with the Risk of Stroke, Heart Disease and All-Cause Mortality

As shown in Table 3, cumulative RCII was positively associated with stroke and all-cause mortality. After full adjustment (Model 3), individuals in the highest cumulative RCII quartile had a 43% higher risk of stroke than those in the lowest quartile (HR: 1.43, 95% CI: 1.00–2.04). For all-cause mortality, the HR for the highest quartile was 2.18 (95% CI: 1.54–3.11), with a PAF of 25.7% (95% CI: 15.1–34.2%). Results for RC and hs-CRP were similar in direction and magnitude (Table S5).

RCS analysis (Figure S6) showed no evidence of a significant non-linear association of cumulative RCII with stroke and heart disease risk (*p* for non-linearity > 0.05). In contrast, a J-shaped non-linear relationship was observed for all-cause mortality (*p* for non-linearity < 0.05), with the lowest hazard ratio at a cumulative RCII level of approximately 10.4. Corresponding analyses for cumulative RC and hs-CRP are shown in Figures S7 and S8.

### 3.4. K-Means Cluster Analysis of RCII Trajectories

Using K-means clustering, participants were classified into four RCII trajectory clusters, with the cluster exhibiting the lowest RCII values designated as the reference group (detailed classification provided in Supplementary Method S2). In the fully adjusted Cox model (Table 4), Cluster 2 showed the highest risk of heart disease (HR = 1.59, 95% CI: 1.16–2.17), with a PAF of 6.3% (95% CI: 2.0–10.4%). Compared with the reference cluster, cluster 3 had a 146% higher risk of all-cause mortality (HR = 2.46, 95% CI: 1.66–3.64), with a progressive trend across clusters (*p* for trend < 0.001). K-means classifications for RC and hs-CRP are presented in Supplementary Table S6.

### 3.5. Subgroup Analysis and Sensitivity Analyses

Subgroup analyses showed an association between RCII and all-cause mortality within the CKM subgroup (*p* = 0.004). A statistically significant interaction between RCII and age was observed for stroke risk (*p* < 0.05). The association was stronger among participants aged <60 years (Table S7). No significant interactions were detected in other subgroups. The main findings were consistent across sensitivity analyses, including exclusion of events occurring early in follow-up, multiple imputation for missing covariates, and alternative categorizations of the exposure variables (Tables S8–S13).

## 4. Discussion

In this study, we investigated the associations between both baseline and cumulative RCII with the risks of stroke, heart disease and all-cause mortality. Our findings suggested that higher RCII levels were associated with higher risks of these outcomes. Participants in the highest RCII quartile had a 55% and a 40% higher risk of developing stroke and heart disease compared with those in the lowest quartile. RCS analysis was consistent with a J-shaped association between cumulative RCII and mortality risk. Moreover, K-means cluster analysis indicated that, relative to the reference group, Cluster 2 (high-to-higher trajectory) had the highest risk of incident heart disease, whereas Cluster 3 (high-to-moderate trajectory) showed the highest risk of all-cause mortality.

The nadir of the J-shaped RCS curve may serve as a risk reference point. This nadir may reflect the lowest combined burden of remnant-lipoprotein cholesterol and low-grade inflammation in participants who are less likely to be affected by frailty or subclinical illness [27]. RCII may be modifiable, as its lipid and inflammatory components could be lowered through lipid-lowering and anti-inflammatory strategies. Evidence is consistent with a causal role of remnant cholesterol in ischemic stroke [13,14,15,16], and anti-inflammatory therapy may reduce recurrent cardiovascular events independent of lipid-lowering [28]. Conversely, higher risk at low RCII may reflect reverse causation or residual confounding (e.g., frailty, undernutrition, or subclinical illness), mirroring U-shaped associations between low LDL-C and higher all-cause mortality reported in a large cohort [27]. In addition, trajectory clusters may capture the combined lipid-inflammation burden over time [29]. Higher and increasing RCII patterns were associated with higher risks, which may indicate potential epidemiological relevance. However, these clusters are data-driven and therefore require external validation and careful interpretation.

Prior epidemiological studies have linked RC to adverse cardiovascular outcomes, including stroke and coronary heart disease [14,15,30], independent of other risk factors [31,32]. Accumulating evidence indicates that evaluating RC and hs-CRP jointly may offer additional prognostic information than either factor alone [33,34], consistent with the concept that lipid-related dysregulation and low-grade inflammation may act together to increase risks of stroke, heart disease, and all-cause mortality [35]. For example, a Danish observational study reported that high RC and hs-CRP in combination corresponded to higher risks of all-cause mortality and stroke than either factor alone [33]. Likewise, findings from a multi-ethnic study of atherosclerosis showed that elevated RC and hs-CRP were linked to a higher risk of atherosclerotic cardiovascular disease (ASCVD) events [34]. Collectively, these findings suggest that jointly assessing both markers may help refine risk stratification for all-cause mortality, heart disease, and stroke. Building on this rationale, we introduced RCII, a composite index intended to characterize the concurrent burden of lipid-related and inflammatory markers in relation to heart disease and stroke. However, evidence on RCII remains relatively limited. A retrospective study reported that RCII predicted unfavorable 3-month outcomes among individuals with stroke, independent of other factors [36]. Furthermore, a cohort study conducted in the United States and China reported that RCII was positively related to all-cause mortality risk among middle-aged and older adults in both countries [37]. In a recent study, Yang et al. examined baseline lnRCII in relation to incident CVD using a composite endpoint among adults with CKM stages 0–3 [25]. By contrast, we evaluated stroke, heart disease, and all-cause mortality separately and incorporated cumulative RCII to characterize longer-term burden, thereby extending the evidence base for RCII-related risk stratification. While prior studies have noted the potential predictive value of RCII, this longitudinal approach may provide complementary and incremental insights into RCII-related risks across CKM stages 0–3.

Our findings show that higher RCII levels are linked to higher risks of stroke, heart disease, and all-cause mortality among adults with CKM stages 0–3. A key feature of the CKM framework, relative to traditional cardiovascular risk factor models, is the explicit inclusion of CKD. The interplay among CKD, metabolic dysfunction, and chronic inflammation may create a self-reinforcing cycle that contributes to disease progression: metabolic disturbances such as ectopic lipid deposition may promote kidney injury through oxidative stress and fibrosis, whereas CKD may further aggravate these abnormalities via toxin accumulation, hormonal dysregulation, and persistent inflammation [38,39]. Although the mechanisms connecting RCII with mortality and cardiovascular outcomes in CKM stages 0–3 are not fully understood, multiple processes could contribute. Inflammation and dyslipidemia may compromise endothelial integrity, reduce nitric oxide bioavailability, disrupt hemostatic balance, and might be linked to accelerated atherosclerosis. Importantly, RCII alone does not necessarily imply a specific biological (e.g., multiplicative) interaction [40,41]. Smoking [42], alcohol consumption [43], obesity [44] and hypertension [45] may also influence stroke and heart disease risk through inflammatory pathways, which we addressed through covariate adjustment. From a prevention perspective, diet may modulate these pathways and may represent a feasible target for intervention. Dietary patterns rich in unsaturated fats, fiber, and polyphenols may improve triglyceride-rich lipoprotein metabolism and attenuate low-grade inflammation, which may contribute to lower RC and RCII [46,47]. A randomized trial reported that Mediterranean diets have beneficial effects on cardiovascular risk factors compared with a low-fat diet [48]. In the PREDIMED trial, a Mediterranean-style diet supplemented with extra-virgin olive oil or nuts was associated with fewer major cardiovascular events [49]. Furthermore, a cholesterol-lowering “portfolio” diet resulted in greater LDL-C reduction than advice to follow a low–saturated-fat diet [50], supporting nutrition as a preventive strategy that may be relevant to early CKM stages.

The sensitivity analyses supported the link between elevated RCII levels, at both baseline and cumulative measures, and higher risk of all-cause mortality, heart disease and stroke. Subgroup analyses indicated that the association between higher RCII levels and stroke risk appeared stronger among participants younger than 60 years. Additionally, we found that the correlation of the index with all-cause mortality risk was more pronounced among CKM patients, a finding supported by related studies [51,52]. A cohort study indicated that higher CKM syndrome stages were associated with increased risks of all-cause and cardiovascular mortality [52].

This study has several notable strengths. First, it focused on individuals in CKM stages 0–3, a clinically relevant yet often underrepresented population. The study extends prior work by evaluating RCII in relation to all-cause mortality, incident heart disease, and incident stroke within CKM stages 0–3, while also considering cumulative RCII and longitudinal patterns, thereby adding evidence relevant to risk assessment in this setting. These findings indicate that RCII may aid early risk assessment and inform management among adults with CKM stages 0–3. Second, the use of nationally representative data from the China Health and Retirement Longitudinal Study supports external validity and statistical reliability. Third, by evaluating baseline, cumulative, and trajectory-based RCII, this study provides a more comprehensive perspective on lipid-inflammation profiles over time and their relation to disease progression.

Nonetheless, several limitations warrant consideration. First, the cohort comprised only middle-aged and older Chinese adults, which may limit generalizability, although the CKM stages 0–3 framework remains a broadly applicable conceptual model. Second, CKM staging relied on the Framingham 10-year CVD risk score rather than the newer PREVENT equation because relevant data were unavailable, which may affect staging accuracy. Third, disease diagnoses were based on self-report, which may lead to underestimation of disease prevalence and potentially bias hazard ratio estimates, and may limit differentiation between specific stroke and heart disease subtypes. Fourth, acute inflammatory responses (e.g., infections) may transiently elevate hs-CRP levels, which could overestimate RCII and attenuate the observed associations. Moreover, RCII (RC × hs-CRP) may overlook additive or other non-multiplicative effects; future studies could explore more rigorous approaches to RCII construction and evaluation. Fifth, despite adjustment for major confounders, residual confounding cannot be excluded, including unmeasured factors such as medication use and detailed lifestyle information. Additionally, mortality ascertainment was restricted to Waves 2–5, and the relatively healthier CKM 0–3 cohort may have contributed to an underestimation of absolute mortality risk. Finally, while our results support an epidemiological association between cumulative RCII and adverse outcomes, the mechanisms underlying inflammation–metabolism interplay and the potential clinical utility of RCII warrant further evaluation, including experimental studies and more rigorous causal inference approaches (e.g., structural equation modeling).

## 5. Conclusions

In conclusion, among individuals with CKM stages 0–3, higher RCII was associated with higher risks of stroke and all-cause mortality. Cumulative RCII showed an even stronger association with all-cause mortality. These findings suggest that RCII may serve as a potential biomarker to help identify individuals at higher risk within CKM stages 0–3, providing a further perspective on the co-occurrence of lipid dysregulation and low-grade inflammation in cardiometabolic health. Future studies could evaluate the clinical utility of incorporating RCII into CKM workflows.

## Figures and Tables

**Table 1:** Baseline characteristics of the study population with and without stroke.

Characteristic	Total (N = 6994)	No (N = 6717)	Yes (N = 277)	*p*
**Age, mean (SD), years**	59.15 (9.32)	59.05 (9.33)	61.67 (8.79)	<0.001
Sex, N (%)				0.858
Female	3660 (52.3)	3517 (52.4)	143 (51.6)	
Male	3334 (47.7)	3200 (47.6)	134 (48.4)	
**Education level, N (%)**				0.312
Illiteracy	1996 (28.5)	1909 (28.4)	87 (31.4)	
Non-illiterate	4998 (71.5)	4808 (71.6)	190 (68.6)	
**Occupation, N (%)**				0.443
Famer	4382 (62.7)	4215 (62.8)	167 (60.3)	
Non-farmer	2612 (37.3)	2502 (37.2)	110 (39.7)	
**Marital Status, N (%)**				0.013
Married	6174 (88.3)	5943 (88.5)	231 (83.4)	
Others	820 (11.7)	774 (11.5)	46 (16.6)	
**Sleep duration, N (%), h/day**				0.422
<7	3503 (50.1)	3358 (50.0)	145 (52.3)	
7–9	3186 (45.6)	3069 (45.7)	117 (42.2)	
≥9	305 (4.4)	290 (4.3)	15 (5.4)	
**Smoke status, N (%)**				0.094
Smoker	2781 (39.8)	2657 (39.6)	124 (44.8)	
Non-smoker	4213 (60.2)	4060 (60.4)	153 (55.2)	
**Drink status, N (%)**				0.402
Yes	5360 (76.6)	5154 (76.7)	206 (74.4)	
No	1634 (23.4)	1563 (23.3)	71 (25.6)	
**SBP, mean (SD), mmHg**	130.60 (21.42)	130.19 (21.19)	140.60 (24.26)	<0.001
**DBP, mean (SD), mmHg**	75.91 (12.12)	75.75 (12.05)	79.87 (13.23)	<0.001
**BMI, kg/m^2^**	23.60 (10.54)	23.56 (10.71)	24.51 (4.93)	0.142
**Cancer, N (%)**				0.468
No	6927 (99.0)	6651 (99.0)	276 (99.6)	
Yes	67 (1.0)	66 (1.0)	1 (0.4)	
**Hypertension, N (%)**				<0.001
No	5554 (79.4)	5378 (80.1)	176 (63.5)	
Yes	1440 (20.6)	1339 (19.9)	101 (36.5)	
**Dyslipidemia treatment, N (%)**				0.138
No	6783 (97.0)	6519 (97.1)	264 (95.3)	
Yes	211 (3.0)	198 (2.9)	13 (4.7)	
**CKM, N (%)**				<0.001
0	669 (9.6)	658 (9.8)	11 (4.0)	
1	1076 (15.4)	1048 (15.6)	28 (10.1)	
2	4663 (66.7)	4457 (66.4)	206 (74.4)	
3	586 (8.4)	554 (8.2)	32 (11.6)	
**RC, mean (SD), mg/dL**	25.94 (24.28)	25.72 (24.04)	31.24 (29.19)	<0.001
**hs-CRP, mean (SD), mg/L**	2.48 (5.71)	2.47 (5.65)	2.66 (7.10)	0.591
**RCII, mean (SD)**	6.20 (13.26)	6.15 (13.14)	7.50 (16.03)	0.096

Abbreviations: SD, standard deviation; BMI, body mass index; SBP, systolic blood pressure; DBP, diastolic blood pressure; CKM, cardiovascular–kidney–metabolic; RC, remnant cholesterol; hs-CRP, high-sensitivity C-reactive protein; RCII, remnant cholesterol inflammatory index.

**Table 2:** Associations of RCII with the risk of stroke, heart disease and all-cause mortality.

Outcome	Events /Total	Model 1			Model 2			Model 3		
		HR (95% CI)	*p*	PAF% (95% CI)	HR (95%CI)	*p*	PAF% (95% CI)	HR (95% CI)	*p*	PAF% (95% CI)
**Stroke**										
Q1	63/2415	Reference	-	-	Reference	-	-	Reference	-	-
Q2	97/2415	1.58 (1.15, 2.17)	0.005	12.8 (4.1, 20.1)	1.53 (1.11, 2.10)	0.009	12.1 (3.2, 19.6)	1.40 (1.02, 1.93)	0.039	10.1 (0.5, 18.3)
Q3	117/2415	1.94 (1.43, 2.63)	<0.001	20.7 (11.9, 28.1)	1.84 (1.35, 2.51)	<0.001	19.2 (10.1, 26.8)	1.55 (1.14, 2.12)	0.006	14.0 (4.3, 22.2)
*p* trend		0.010			0.029			0.013		
**Heart disease**										
Q1	115/2332	Reference	-	-	Reference	-	-	Reference	-	-
Q2	160/2331	1.43 (1.13, 1.82)	0.003	10.8 (3.8, 17.1)	1.42 (1.11, 1.80)	0.005	10.6 (3.4, 17.0)	1.36 (1.07, 1.74)	0.012	9.6 (2.2, 16.2)
Q3	172/2331	1.56 (1.23, 1.98)	<0.001	14.0 (6.9, 20.3)	1.51 (1.19, 1.92)	<0.001	13.0 (5.8, 19.5)	1.40 (1.10, 1.79)	0.006	10.7 (3.2, 17.4)
*p* trend		0.086			0.130			0.230		
**All-cause mortality**										
Q1	166/2415	Reference	-	-	Reference	-	-	Reference	-	-
Q2	210/2415	1.30 (1.06, 1.59)	0.012	7.3 (1.6, 12.5)	1.26 (1.02, 1.54)	0.029	6.6 (0.6, 12.1)	1.30 (1.06, 1.59)	0.013	7.4 (1.5, 12.8)
Q3	277/2415	1.73 (1.43, 2.10)	<0.001	18.1 (12.2, 23.4)	1.56 (1.29, 1.90)	<0.001	14.6 (8.5, 20.0)	1.67 (1.37, 2.04)	<0.001	16.6 (10.5, 22.0)
*p* trend		0.012			0.029			0.013		

Abbreviations: RCII, remnant cholesterol inflammatory index; PAF, population attributable fraction; HR, hazard ratio; CI, confidence interval; Q1, quantile 1; Q2, quantile 2; Q3, quantile 3; SBP, systolic blood pressure; DBP, diastolic blood pressure; BMI, body mass index. Model 1: Unadjusted; Model 2: Adjusted age, sex, education level, occupation, marital Status, sleep duration, smoke status, drink status; Model 3: Adjusted age, sex, education level, occupation, marital Status, sleep duration, smoke status, drink status, SBP, DBP, BMI, cancer, hypertension, dyslipidemia treatment.

**Table 3:** Associations of cumulative RCII with the risk of stroke, heart disease and all-cause mortality.

Outcome	Events /Total	Model 1			Model 2			Model 3		
		HR (95% CI)	*p*	PAF% (95% CI)	HR (95% CI)	*p*	PAF% (95% CI)	HR (95%CI)	*p*	PAF% (95% CI)
**Stroke**										
Q1	51/1569	Reference	-	-	Reference	-	-	Reference	-	-
Q2	73/1569	1.45 (1.01, 2.07)	0.043	10.8 (0.3, 19.6)	1.41 (0.99, 2.02)	0.060	10.3 (−0.5, 19.3)	1.35 (0.94, 1.93)	0.102	9.2 (−2.0, 18.7)
Q3	84/1569	1.67 (1.18, 2.37)	0.004	16.3 (5.6, 25.1)	1.60 (1.12, 2.27)	0.009	14.9 (4.0, 24.0)	1.43 (1.00, 2.04)	0.047	11.4 (0.1, 21.0)
*p* trend		0.043			0.060			0.102		
**Heart diseases**										
Q1	96/1569	Reference	-	-	Reference	-	-	Reference	-	-
Q2	116/1569	1.21 (0.93, 1.59)	0.159	5.9 (−2.4, 13.3)	1.19 (0.91, 1.57)	0.201	5.4 (−3.0, 12.9)	1.16 (0.88, 1.52)	0.294	4.6 (−4.1, 12.3)
Q3	135/1569	1.43 (1.10, 1.86)	0.008	11.7 (3.3, 19.2)	1.40 (1.07, 1.82)	0.013	11.1 (2.5, 18.7)	1.29 (0.99, 1.69)	0.060	8.5 (−0.4, 16.4)
*p* trend		0.159			0.201			0.293		
**All-cause mortality**										
Q1	48/1633	Reference	-	-	Reference	-	-	Reference	-	-
Q2	62/1633	1.3 (0.89, 1.89)	0.178	6.4 (−3.2, 14.5)	1.34 (0.91, 1.96)	0.133	7.4 (−2.8, 15.9)	1.37 (0.93, 2.01)	0.110	7.8 (−2.3, 16.2)
Q3	108/1633	2.3 (1.64, 3.23)	<0.001	28.2 (17.9, 36.3)	2.12 (1.50, 3.00)	<0.001	24.9 (14.3, 33.3)	2.18 (1.54, 3.11)	<0.001	25.7 (15.1, 34.2)
*p* trend		0.178			0.133			0.110		

Abbreviations: RCII, remnant cholesterol inflammatory index; PAF, population attributable fraction; HR, hazard ratio; CI, confidence interval; Q1, quantile 1; Q2, quantile 2; Q3, quantile 3; SBP, systolic blood pressure; DBP, diastolic blood pressure; BMI, body mass index. Model 1: Unadjusted; Model 2: Adjusted age, sex, education level, occupation, marital Status, sleep duration, smoke status, drink status; Model 3: Adjusted age, sex, education level, occupation, marital Status, sleep duration, smoke status, drink status, SBP, DBP, BMI, cancer, hypertension, dyslipidemia treatment.

**Table 4:** Associations of K-means RCII with the risk of stroke, heart disease and all-cause mortality.

Outcome	Events /Total	Model 1			Model 2			Model 3		
		HR (95%CI)	*p*	PAF% (95% CI)	HR (95%CI)	*p*	PAF% (95% CI)	HR (95%CI)	*p*	PAF% (95% CI)
**Stroke**										
Cluster 1	55/961	1.58 (1.12, 2.24)	0.010	9.8 (2.4, 16.2)	1.50 (1.06, 2.12)	0.023	8.6 (1.2, 15.1)	1.40 (0.98, 1.98)	0.062	7.2 (−0.4, 14.0)
Cluster 2	36/590	1.69 (1.14, 2.51)	0.010	7.1 (1.8, 12.2)	1.59 (1.06, 2.37)	0.024	6.2 (0.8, 11.3)	1.36 (0.91, 2.05)	0.136	4.1 (−1.3, 9.2)
Cluster 3	76/2071	Reference	-	-	Reference	-	-	Reference	-	-
Cluster 4	41/1085	1.03 (0.70, 1.51)	0.880	0.6 (−7.0, 7.5)	1.03 (0.7, 1.5)	0.896	0.5 (−7.3, 7.6)	0.97 (0.66, 1.43)	0.893	−0.6 (−8.6, 6.9)
*p* trend		0.010			0.023			0.061		
**Heart diseases**										
Cluster1	80/961	1.37 (1.04, 1.81)	0.027	6.2 (0.7, 11.3)	1.36 (1.03, 1.81)	0.032	6.1 (0.5, 11.2)	1.30 (0.98, 1.72)	0.073	5.2 (−0.5, 10.5)
Cluster2	62/590	1.76 (1.30, 2.39)	<0.001	7.9 (3.7, 11.8)	1.74 (1.28, 2.37)	<0.001	7.7 (3.4, 11.7)	1.59 (1.16, 2.17)	0.004	6.3 (2.0, 10.4)
Cluster3	127/2071	Reference	-	-	Reference	-	-	Reference	-	-
Cluster4	78/1085	1.18 (0.89, 1.57)	0.247	3.4 (−2.5, 8.9)	1.17 (0.88, 1.55)	0.288	3.2 (−2.8, 8.6)	1.12 (0.84, 1.49)	0.428	2.4 (−3.7, 8.1)
*p* trend		0.027			0.032			0.073		
**All-cause mortality**										
Cluster 1	64/1124	1.94 (1.37, 2.74)	<0.001	14.1 (7.1, 20.0)	1.91 (1.35, 2.72)	<0.001	14.3 (6.8, 20.6)	1.97 (1.38, 2.80)	<0.001	14.6 (7.2, 20.8)
Cluster 2	43/1013	1.43 (0.97, 2.11)	0.069	5.9 (−0.5, 11.5)	1.32 (0.89, 1.95)	0.164	4.5 (−2.0, 10.3)	1.34 (0.90, 1.99)	0.144	4.7 (−1.8, 10.4)
Cluster 3	48/639	2.61 (1.79, 3.80)	<0.001	13.8 (8.7, 18.2)	2.29 (1.56, 3.35)	<0.001	11.4 (6.2, 15.9)	2.46 (1.66, 3.64)	<0.001	12.6 (7.2, 17.3)
Cluster 4	63/2123	Reference	-	-	Reference	-	-	Reference	-	-
*p* trend		<0.001			<0.001			<0.001		

Abbreviations: RCII, remnant cholesterol inflammatory index; PAF, population attributable fraction; HR, hazard ratio; CI, confidence interval; SBP, systolic blood pressure; DBP, diastolic blood pressure; BMI, body mass index. Model 1: Unadjusted; Model 2: Adjusted age, sex, education level, occupation, marital Status, sleep duration, smoke status, drink status; Model 3: Adjusted age, sex, education level, occupation, marital Status, sleep duration, smoke status, drink status, SBP, DBP, BMI, cancer, hypertension, dyslipidemia treatment.

## Data Availability

The datasets generated and analyzed during the current study are available on the CHARLS website, available at http://charls.pku.edu.cn/en (accessed on 12 April 2024).

## Supplementary Materials

The following supporting information can be downloaded at: https://www.mdpi.com/article/10.3390/nu18020205/s1, Method S1: Definition of Subclinical Cardiovascular Disease (CVD) and Cardiometabolic Health (CKM) stages 0–3; Method S2: K-means Clustering of RCII, RC and hs-CRP Distribution Patterns; Figure S1: Kaplan–Meier curves of stroke (A), heart disease (B) and all-cause mortality (C) according to the quartiles of the RCII in participants with CKM syndrome stages 0–3; Figure S2: Kaplan–Meier curves of stroke (A), heart disease (B) and all-cause mortality (C) according to the quartiles of the RC in participants with CKM syndrome stages 0–3; Figure S3: Kaplan–Meier curves of stroke (A), heart disease (B) and all-cause mortality (C) according to the quartiles of the hs-CRP in participants with CKM syndrome stages 0–3; Figure S4: RCS analysis of the association of RC with stroke (A), heart disease (B) and all-cause mortality (C) in individuals with CKM syndrome stages 0–3; Figure S5: RCS analysis of the association of hs-CRP with stroke (A), heart disease (B) and all-cause mortality (C) in individuals with CKM syndrome stages 0–3; Figure S6: RCS analysis of the association of cumulative RCII with stroke (A), heart disease (B) and all-cause mortality (C) in individuals with CKM syndrome stages 0–3; Figure S7: RCS analysis of the association of cumulative RC with stroke (A), heart disease (B) and all-cause mortality (C) in individuals with CKM syndrome stages 0–3; Figure S8: RCS analysis of the association of cumulative hs-CRP with stroke (A), heart disease (B) and all-cause mortality (C) in individuals with CKM syndrome stages 0–3; Figure S9: Receiver operating characteristic (ROC) curves comparing the discriminative performance of RCII, RC and hs-CRP for incident stroke (A), heart disease (B) and all-cause mortality (C) in individuals with CKM syndrome stages 0–3; Figure S10: Receiver operating characteristic (ROC) curves comparing the discriminative performance of cumulative RCII, cumulative RC and cumulative hs-CRP for incident stroke (A), all-cause mortality (B) and heart disease (C) in individuals with CKM syndrome stages 0–3; Table S1: Baseline characteristics of the study population with and without heart disease; Table S2: Baseline characteristics of the study individuals in death; Table S3: Baseline characteristics of the study population with and without all-cause death; Table S4: Associations of RC and hs-CRP with the risk of stroke, heart disease and all-cause mortality; Table S5: Associations of cumulative RC and cumulative CRP with the risk of stroke, heart disease and all-cause mortality; Table S6: Associations of K-means RC and K-means CRP with the risk of stroke, heart disease and all-cause mortality; Table S7: Subgroup Analysis of RCII with the risk of stroke, heart disease and all-cause mortality; Table S8: Multivariable Cox regression analysis of RCII, RC and hs-CRP association with the risk of stroke, heart disease and all-cause mortality in individuals with CKM syndrome (stages 0–3): after excluding individuals with missing covariates; Table S9: Multivariable Cox regression analysis of RCII, RC and hs-CRP association with the risk of stroke, heart disease and all-cause mortality in individuals with CKM syndrome (stages 0–3): after excluding participants who was observed the outcomes within 2 years of baseline; Table S10: Associations of RCII, RC and hs-CRP with the risk of stroke, heart disease and all-cause mortality by using age as the time scale; Table S11: Multivariable Cox regression analysis of RCII, RC and hs-CRP association with the risk of stroke, heart disease and all-cause mortality in individuals with CKM syndrome (stages 0–3): using dichotomized exposure variable; Table S12: Multivariable Cox regression analysis of RCII, RC and hs-CRP association with the risk of stroke, heart disease and all-cause mortality in individuals with CKM syndrome (stages 0–3): using quartiles exposure variable; Table S13: Multivariable Cox regression analysis of RCII, RC and hs-CRP association with the risk of stroke, heart disease and all-cause mortality in individuals with CKM syndrome (stages 0–3): using quintiles exposure variable.

## Abbreviations

The following abbreviations are used in this manuscript:

CKM	Cardiovascular–kidney–metabolic
CVD	Cardiovascular disease
ASCVD	Atherosclerotic cardiovascular disease
CKD	Chronic kidney disease
RCII	Remnant cholesterol inflammatory index
RC	Remnant cholesterol
hs-CRP	High-sensitivity C-reactive protein
TC	Total cholesterol
HDL-C	High-density lipoprotein cholesterol
LDL-C	Low-density lipoprotein cholesterol
cuRCII	Cumulative Remnant cholesterol inflammatory index
BMI	Body Mass Index
SBP	Systolic blood pressure
DBP	Diastolic blood pressure
CHARLS	China Health and Retirement Longitudinal Study
AHA	American Heart Association
IRB	Institutional Review Board
IQR	Interquartile range
RCS	Restricted cubic spline
PAF	Population attributable fraction
HR	Hazard ratio
CI	Confidence interval
