# Supplementary material for: Association of Remnant Cholesterol Inflammatory Index with Stroke, Heart Disease and All-Cause Mortality Across Cardiovascular–Kidney–Metabolic Syndrome Stages 0–3: A National Cohort Study
Source: Nutrients. 2026 Jan 8;18(2):205. doi: 10.3390/nu18020205 (PMC12844947; doi:10.3390/nu18020205)
Supplement: Supplementary file 1 [file nutrients-18-00205-s001.zip › nutrients-4036526-supplementary.pdf]

Association of Remnant Cholesterol Inflammatory Index with Stroke, Heart Disease and All-Cause Mortality across Cardiovascular–Kidney–Metabolic Syndrome Stages 0–3: A National Cohort Study

Supplementary content

Method S1 Definition of Subclinical Cardiovascular Disease (CVD) and Cardiometabolic Health (CKM) Stages 0–3. 3
Method S2 K-means Clustering of RCII, RC and hs-CRP Distribution Patterns. 6
Figure S1 Kaplan–Meier curves of stroke (A), heart disease (B) and all-cause mortality (C) according to the quartiles of the RCII in participants with CKM syndrome stages 0–3. 11
Figure S2 Kaplan–Meier curves of stroke (A), heart disease (B) and all-cause mortality (C) according to the quartiles of the RC in participants with CKM syndrome stages 0–3 12
Figure S3 Kaplan–Meier curves of stroke (A), heart disease (B) and all-cause mortality (C) according to the quartiles of the hs-CRP in participants with CKM syndrome stages 0–3 13
Figure S4 RCS analysis of the association of RC with stroke (A), heart disease (B) and all-cause mortality (C) in individuals with CKM syndrome stages 0–3. 14
Figure S5 RCS analysis of the association of hs-CRP with stroke (A), heart disease (B) and all-cause mortality (C) in individuals with CKM syndrome stages 0–3. 15
Figure S6 RCS analysis of the association of cumulative RCII with stroke (A), heart disease (B) and all-cause mortality (C) in individuals with CKM syndrome stages 0–3. 16
Figure S7 RCS analysis of the association of cumulative RC with stroke (A), heart disease (B) and all-cause mortality (C) in individuals with CKM syndrome stages 0–3. 17
Figure S8 RCS analysis of the association of cumulative hs-CRP with stroke (A), heart disease (B) and all-cause mortality (C) in individuals with CKM syndrome stages 0–3. 18
Figure S9 Receiver operating characteristic (ROC) curves comparing the discriminative performance of RCII, RC and hs-CRP for incident stroke (A), heart disease (B) and all-cause mortality (C) in individuals with CKM syndrome stages 0–3. 19
Figure S10 Receiver operating characteristic (ROC) curves comparing the discriminative performance of cumulative RCII, cumulative RC and cumulative hs-CRP for incident stroke (A), all-cause mortality (B) and heart disease (C) in individuals with CKM syndrome stages 0–3 20
Table S1. Baseline characteristics of the study population with and without heart disease. 21
Table S2. Baseline characteristics of the study individuals in death. 23
Table S3. Baseline characteristics of the study population with and without all-cause death 25
Table S4. Associations of RC and hs-CRP with the risk of stroke, heart disease and all-cause mortality. 27
Table S5. Associations of cumulative RC and cumulative CRP with the risk of stroke, heart disease and all-cause mortality. 29
Table S6. Associations of K-means RC and K-means CRP with the risk of stroke, heart disease and all-cause mortality. 31
Table S7. Subgroup Analysis of RCII with the risk of stroke, heart disease and all-cause mortality. 33

Table S8. Multivariable Cox regression analysis of RCII, RC and hs-CRP association with the risk of stroke, heart disease and all-cause mortality in individuals with CKM syndrome (stages 0–3): after excluding individuals with missing covariates. 37
Table S9. Multivariable Cox regression analysis of RCII, RC and hs-CRP association with the risk of stroke, heart disease and all-cause mortality in individuals with CKM syndrome (stages 0–3): after excluding participants who was observed the outcomes within 2 years of baseline..............39
Table S10. Associations of RCII, RC and hs-CRP with the risk of stroke, heart disease and all-cause mortality by using age as the time scale. 42
Table S11. Multivariable Cox regression analysis of RCII, RC and hs-CRP association with the risk of stroke, heart disease and all-cause mortality in individuals with CKM syndrome (stages 0–3): using dichotomized exposure variable..44
Table S12. Multivariable Cox regression analysis of RCII, RC and hs-CRP association with the risk of stroke, heart disease and all-cause mortality in individuals with CKM syndrome (stages 0–3): using quartiles exposure variable..46
Table S13. Multivariable Cox regression analysis of RCII, RC and hs-CRP association with the risk of stroke, heart disease and all-cause mortality in individuals with CKM syndrome (stages 0–3): using quintiles exposure variable..49

Method S1 Definition of Subclinical Cardiovascular Disease (CVD) and Cardiometabolic Health (CKM) Stages 0–3.

Definition of Subclinical Cardiovascular Disease (CVD)

Subclinical CVD was defined as either (1) very high-risk chronic kidney disease (CKD) according to the Kidney Disease: Improving Global Outcomes (KDIGO) classification (estimated glomerular filtration rate [eGFR] <30 mL/min/1.73m^2), or (2) a Framingham risk score ≥21.5 for women or ≥21.6 for men.

	Points	Age, years	HDL	TC	SBP Not Treated	SBP Treated	Smoker	Diabetic
**Male**	-2		60+		<120			
	-1		50-59					
	0	30-34	45-49	<160	120-129	<120	No	No
	1		35-44	160-199	130-139			
	2	35-39	<35	200-239	140-159	120-129		
	3			240-279	160+	130-139		Yes
	4			280+		140-159	Yes	
	5	40-44				160+		
	6	45-49						
	7							
	8	50-54						
	9							
	10	55-59						
	11	60-64						
	12	65-69						
	13							
	14	70-74						
	15	75+						
**Female**	-3				<120			
	-2		60+					
	-1		50-59			<120		
	0	30-34	45-49	<160	120-129		No	No
	1		35-44	160-199	130-139			
	2	35-39	<35		140-149	120-129		
	3			200-239		130-139	Yes	
	4	40-44		240-279	150-159			Yes
	5	45-49		280+	160+	140-149		
	6					150-159		
	7	50-54				160+		
	8	55-59						
	9	60-64						
	10	65-69						

11	70-74
12	75+

Definition of Cardiometabolic Health (CKM) Stages 0–3

Participants were classified into CKM stages based on anthropometric, metabolic, and renal function indicators, as well as the presence of clinical or subclinical CVD.

Stage 0:

All of the following criteria were met:

(1) body mass index (BMI) <23 kg/m²;

(2) waist circumference <80 cm for women and <90 cm for men;

(3) fasting blood glucose <100 mg/dL and glycated hemoglobin (HbA1c) <5.7%, without a self-reported diagnosis of diabetes or use of glucose-lowering medication;

(4) systolic blood pressure (SBP) <130 mmHg and diastolic blood pressure (DBP) <80 mmHg, without a self-reported diagnosis of hypertension or use of antihypertensive medication;

(5) high-density lipoprotein cholesterol (HDL-C) <50 mg/dL for women and <40 mg/dL for men;

(6) triglycerides <150 mg/dL;

(7) eGFR ≥60 mL/min/1.73m², without a self-reported diagnosis of CKD;

(8) no clinical or subclinical CVD.

Stage 1:

Presence of at least one of the following: overweight/obesity, abdominal obesity, or prediabetes. All of the following must also be met: normal blood pressure, HDL-C within the healthy range, triglycerides <150 mg/dL, eGFR ≥60 mL/min/1.73m² without CKD, and no clinical or subclinical CVD.

Stage 2:

Presence of at least one of the following: hypertriglyceridemia, hypertension, diabetes, metabolic syndrome, or eGFR between 30-60 mL/min/1.73m² (or a self-reported diagnosis of CKD). Participants must have no clinical or subclinical CVD.

Stage 3:

Presence of either eGFR <30 mL/min/1.73m^2 or subclinical CVD, along with at least one of the following: overweight/obesity, abdominal obesity, prediabetes, hypertriglyceridemia, hypertension, diabetes, metabolic syndrome, or eGFR between 30-60 mL/min/1.73m^2. Participants must have no clinical CVD.

Method S2 K-means Clustering of RCII, RC and hs-CRP Distribution Patterns.

In this study, we performed K-means clustering to identify potential distribution patterns of three indicators—RCII, RC, and hs-CRP—in relation to CVD outcomes. The clustering analysis was conducted in R using the "cluster" and "factoextra" packages. The optimal number of clusters was determined to be four based on the elbow method. Each sample was then assigned to the nearest cluster center, and the cluster centers were iteratively updated until convergence or a predefined number of iterations was reached.

(1) Stroke

- RCII: Class 1 (low-decreasing, range 10.12–4.25; 5.7%), Class 2 (high-increasing, range 22.33–30.98; 6.1%), Class 3 (persistently low, range 1.10–1.91; 3.7%), and Class 4 (low-increasing, range 2.03–12.91; 3.8%).

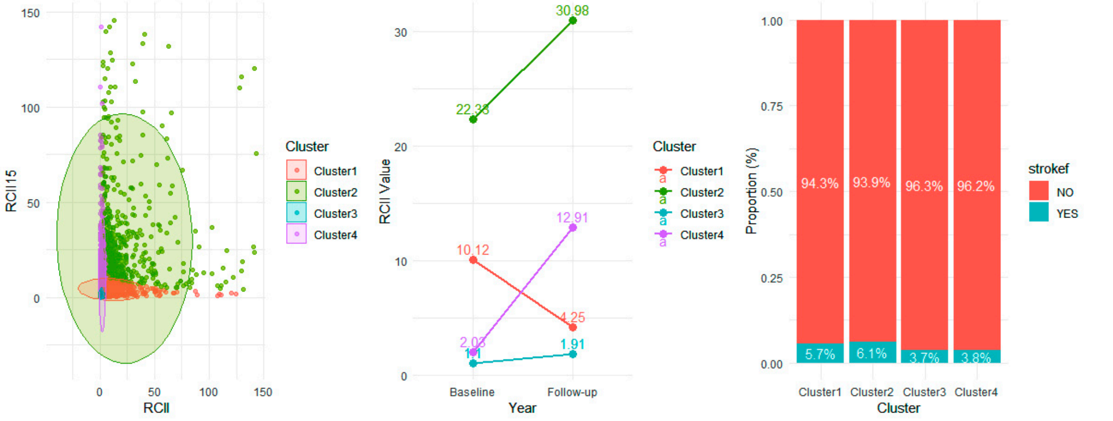

- RC: Class 1 (low-to-high, 6.83–22.90; 3.2%), Class 2 (persistently high, 58.53–57.45; 5.0%), Class 3 (medium-to-high, 22.35–31.92; 5.2%), and Class 4 (high-to-medium, 22.02–17.10; 4.0%).

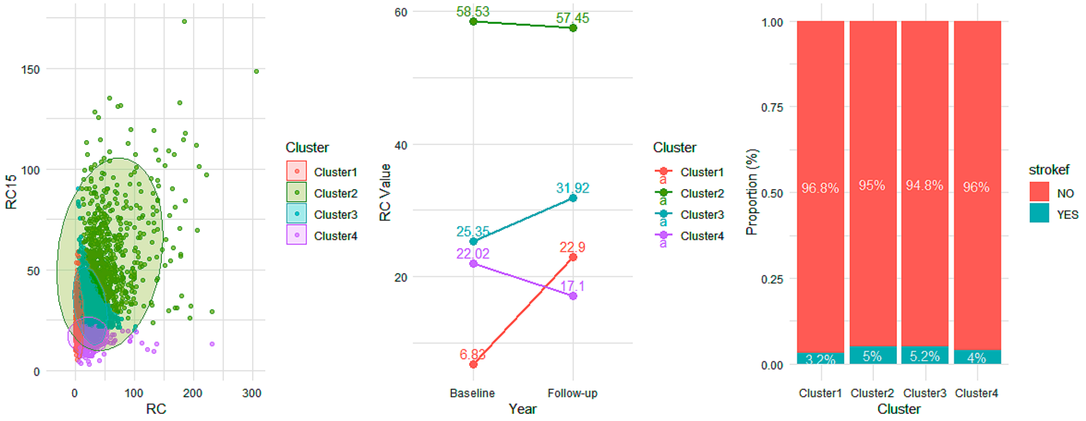

- hs-CRP: Class 1 (low-to-high; 2.23–10.59, 4.1%), Class 2 (persistently low, 0.68–0.78; 3.8%), Class 3 (persistently moderate, 1.64–2.22; 5.0%), and Class 4 (moderate-to-high, 2.33–10.59; 5.7%).

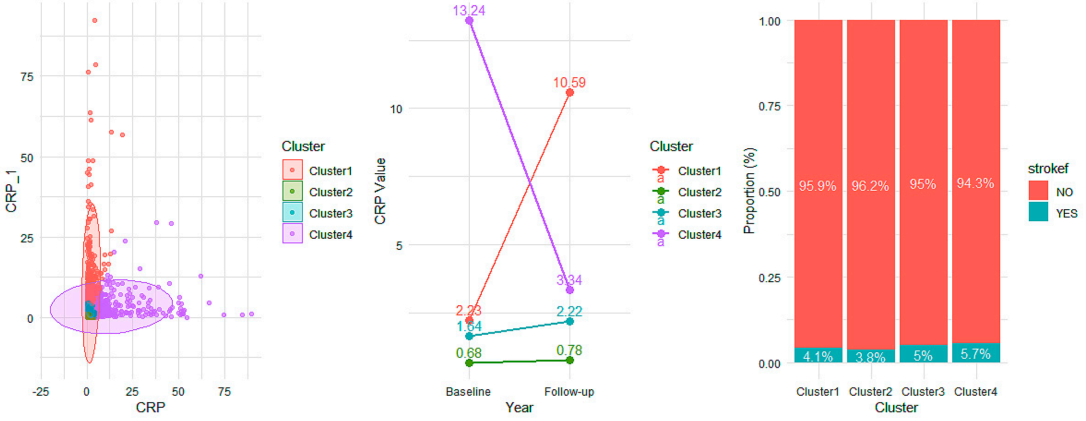

(2) Heart disease

- RCII: Class 1 (low-to-lower, 10.12–4.25; 8.3%), Class 2 (high-to-higher, 22.33–30.98; 10.5%), Class 3 (consistently low, 1.10–1.91; 6.1%), and Class 4 (low-to-high, 2.03–12.91; 7.2%).

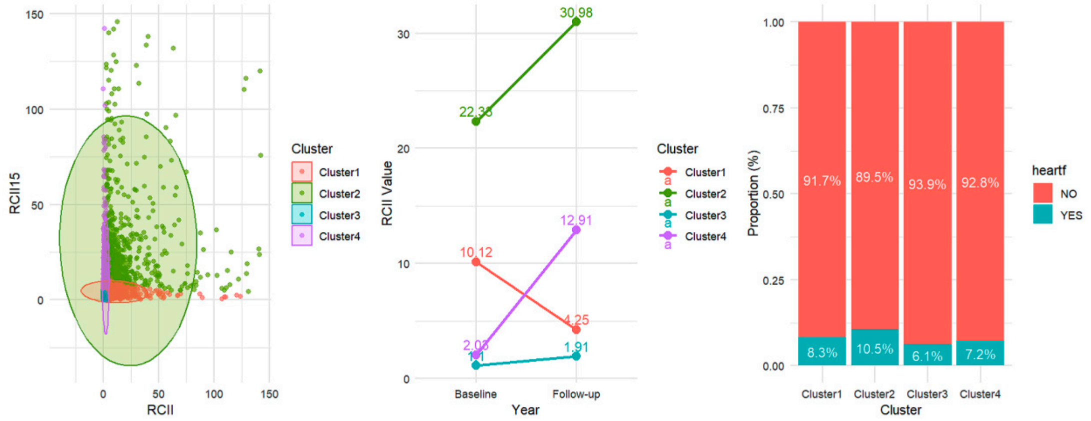

- RC: Class 1 (low-to-high, 6.83–22.90; 6.6%), Class 2 (consistently high, 58.53–57.45; 8.6%), Class 3 (moderate-to-high, 22.35–31.92; 7.9%), and Class 4 (high-to-moderate, 22.02–17.10; 6.4%).

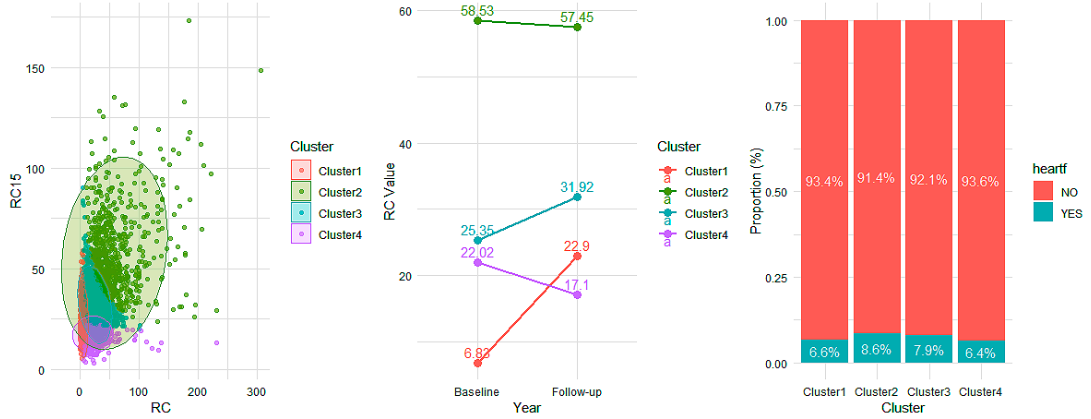

- hs-CRP: Class 1 (low-to-high, 2.23–10.59; 9.1%), Class 2 (consistently low, 0.68–0.78; 6.5%), Class 3 (consistently moderate, 1.64–2.22; 7.3%), and Class 4 (moderate-to-high, 2.33–10.59; 10.4%).

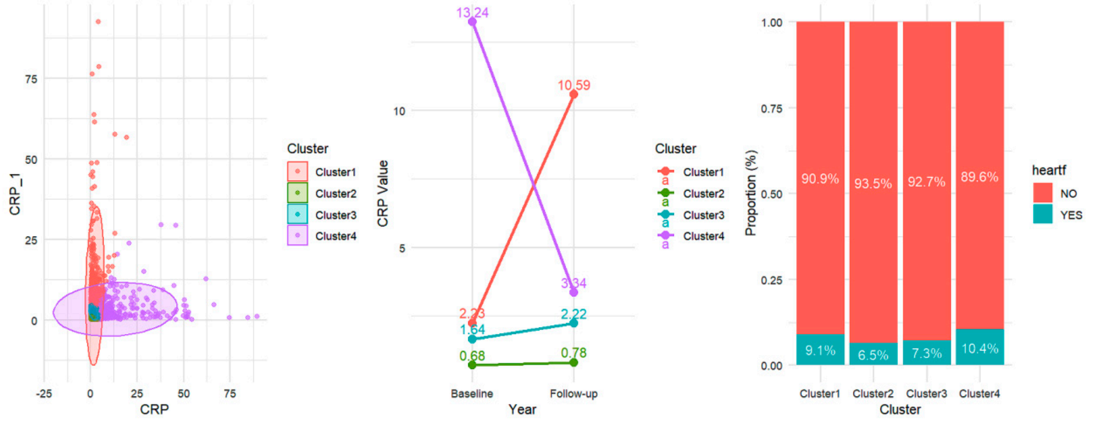

All-cause mortality

- RCII: Class 1 (low-to-high, 2.01–13.03; 5.7%), Class 2 (high-to-low, 9.60–4.14; 4.2%), Class 3 (high-to-higher, 21.65–29.26; 7.5%), and Class 4 (consistently low, 1.09–1.91; 3.0%).

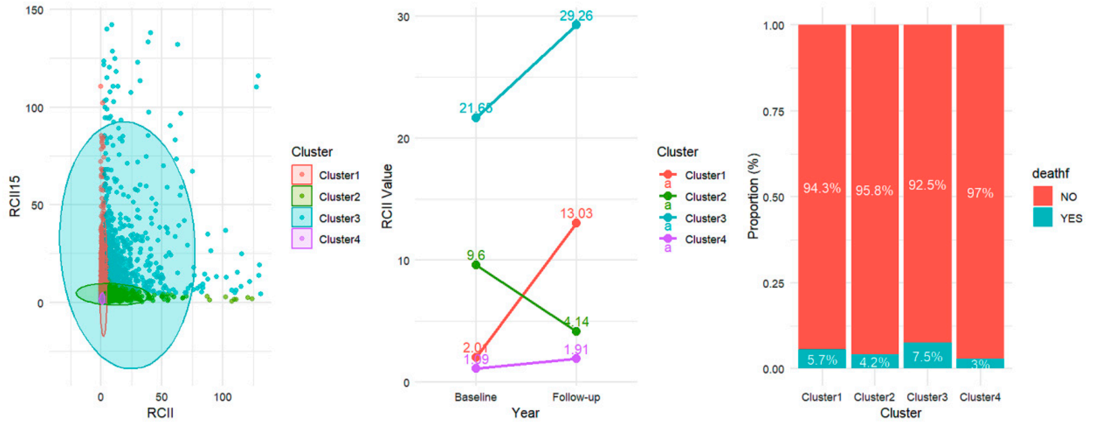

- RC: Class 1 (consistently high, 57.39–56.93; 4.2%), Class 2 (high-to-moderate, 22.03–17.12; 4.6%), Class 3 (low-to-high, 6.77–23.02; 4.5%), and Class 4 (consistently moderate, 25.39–31.80; 4.4%).

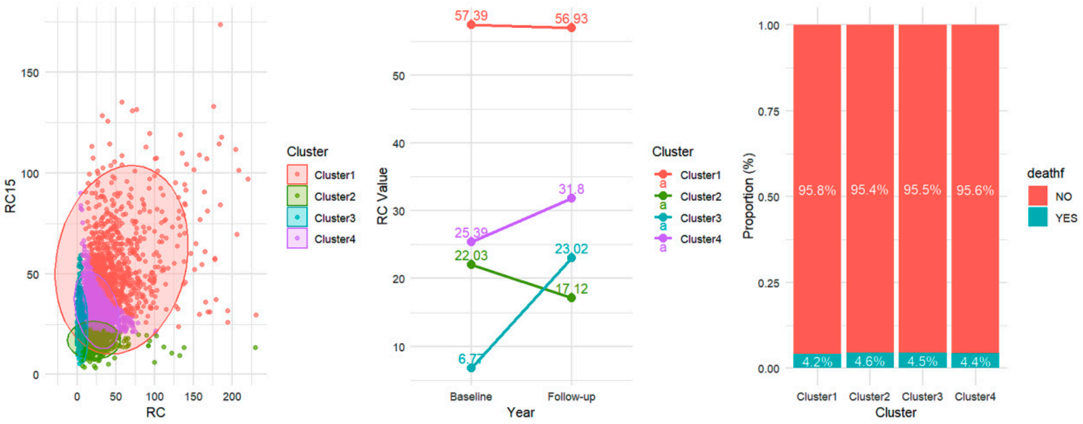

- hs-CRP: Class 1 (low-to-moderate, 1.62–2.21; 3.9%), Class 2 (high-to-low, 12.66–3.22; 6.5%), Class 3 (low-to-high, 2.27–10.50; 10.8%), and Class 4 (consistently low, 0.69–0.78; 3.0%).

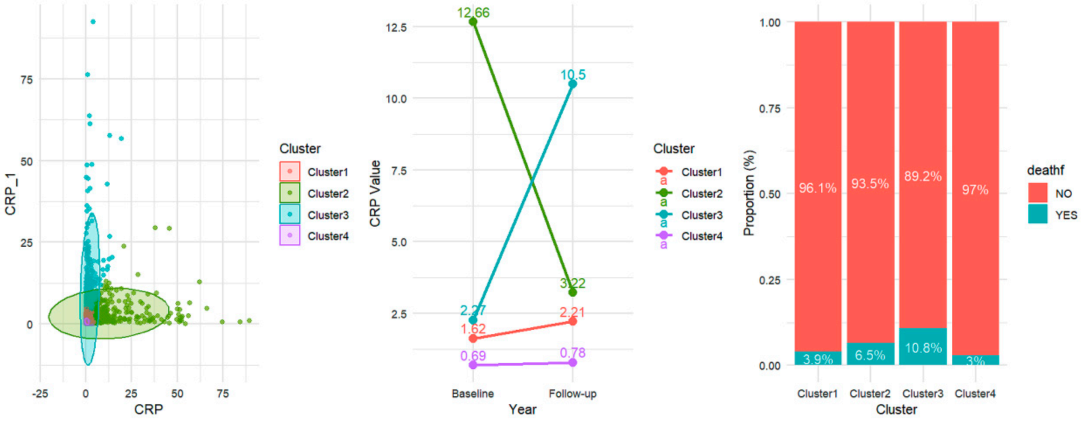

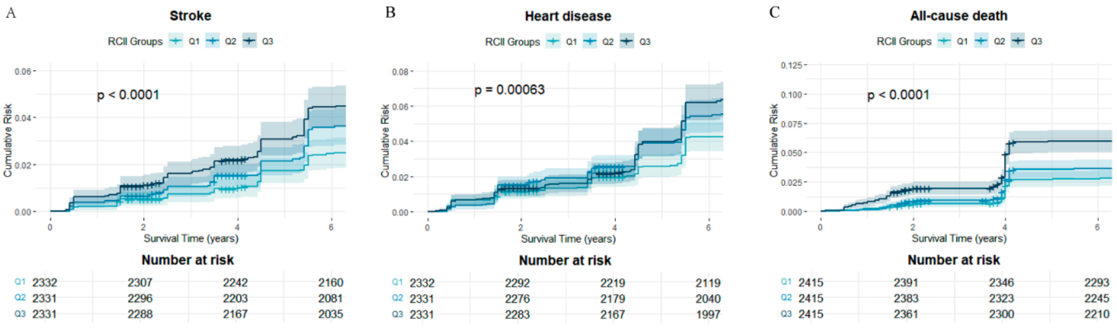

Figure S1 Kaplan–Meier curves of stroke (A), heart disease (B) and all-cause mortality (C) according to the quartiles of the RCII in participants with CKM syndrome stages 0–3.

Adjusted age, sex, education level, occupation, marital Status, sleep duration, smoke status, drink status, SBP, DBP, BMI, cancer, hypertension, dyslipidemia treatment.

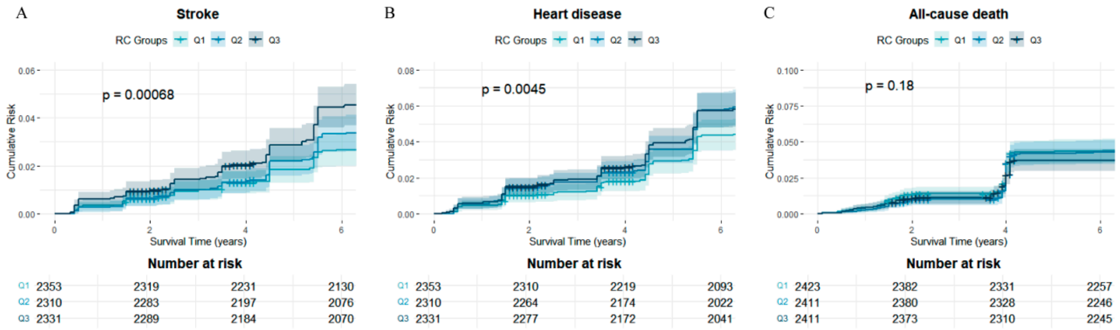

Figure S2 Kaplan–Meier curves of stroke (A), heart disease (B) and all-cause mortality (C) according to the quartiles of the RC in participants with CKM syndrome stages 0–3.

Adjusted age, sex, education level, occupation, marital Status, sleep duration, smoke status, drink status, SBP, DBP, BMI, cancer, hypertension, dyslipidemia treatment.

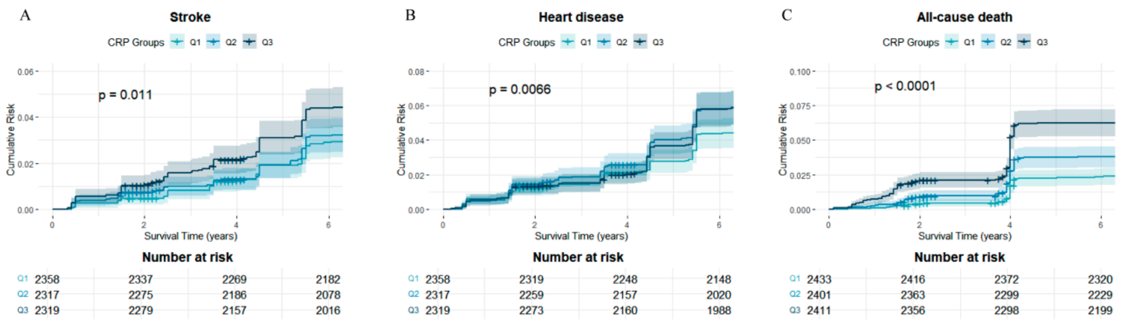

Figure S3 Kaplan–Meier curves of stroke (A), heart disease (B) and all-cause mortality (C) according to the quartiles of the hs-CRP in participants with CKM syndrome stages 0–3.

Adjusted age, sex, education level, occupation, marital Status, sleep duration, smoke status, drink status, SBP, DBP, BMI, cancer, hypertension, dyslipidemia treatment.

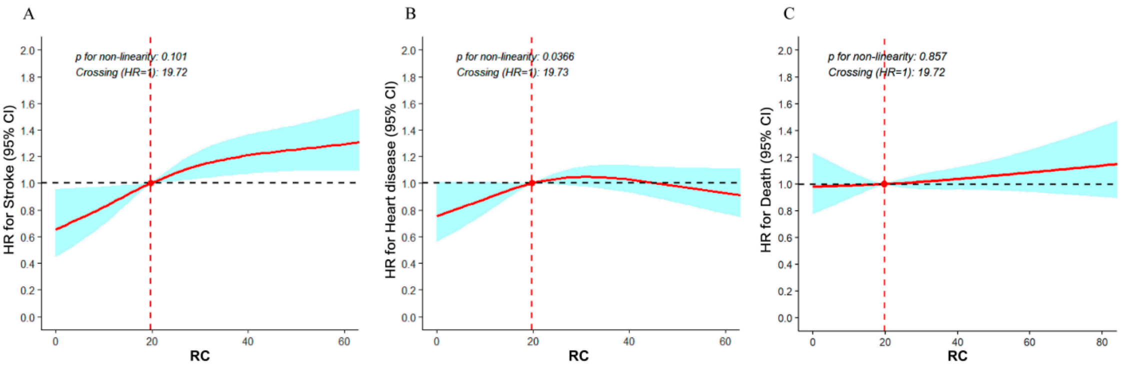

Figure S4 RCS analysis of the association of RC with stroke (A), heart disease (B) and all-cause mortality (C) in individuals with CKM syndrome stages 0–3.

Adjusted age, sex, education level, occupation, marital Status, sleep duration, smoke status, drink status, SBP, DBP, BMI, cancer, hypertension, dyslipidemia treatment.

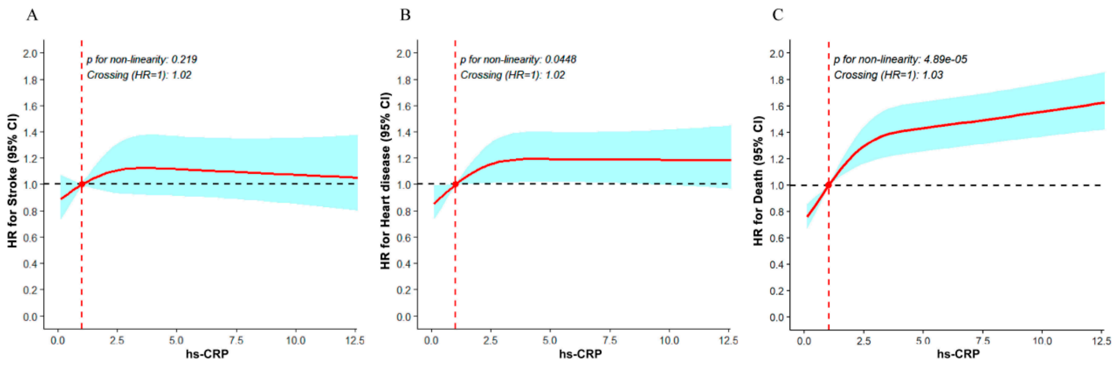

Figure S5 RCS analysis of the association of hs-CRP with stroke (A), heart disease (B) and all-cause mortality (C) in individuals with CKM syndrome stages 0–3.

Adjusted age, sex, education level, occupation, marital Status, sleep duration, smoke status, drink status, SBP, DBP, BMI, cancer, hypertension, dyslipidemia treatment.

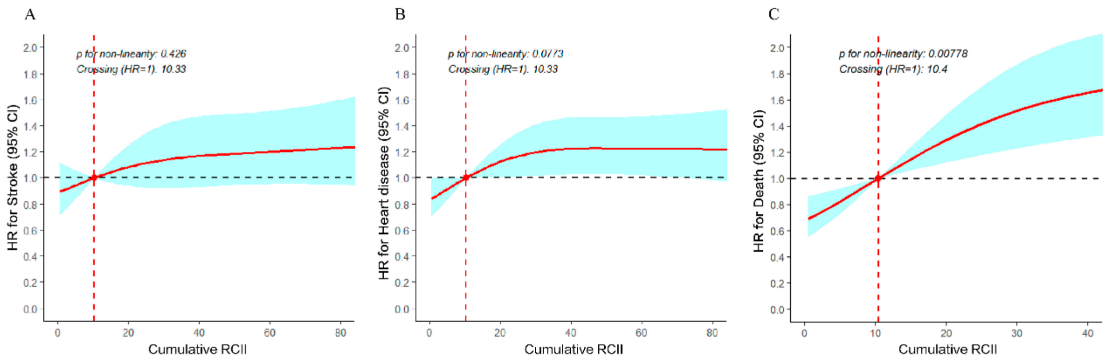

Figure S6 RCS analysis of the association of cumulative RCII with stroke (A), heart disease (B) and all-cause mortality (C) in individuals with CKM syndrome stages 0–3.

Adjusted age, sex, education level, occupation, marital Status, sleep duration, smoke status, drink status, SBP, DBP, BMI, cancer, hypertension, dyslipidemia treatment.

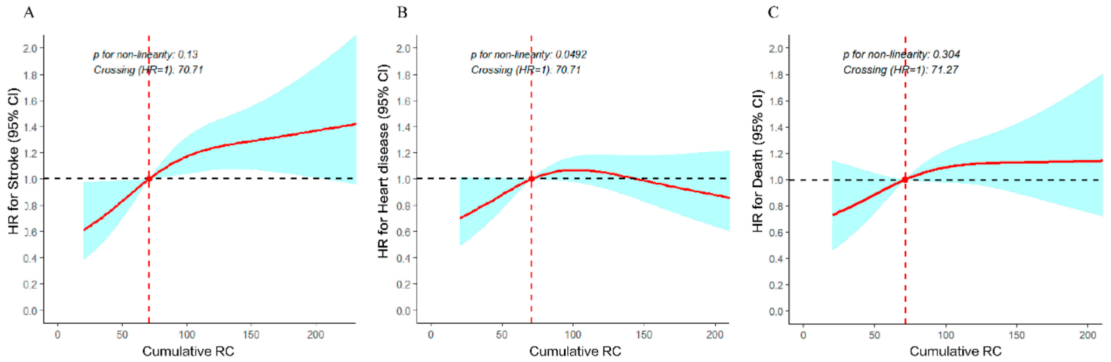

Figure S7 RCS analysis of the association of cumulative RC with stroke (A), heart disease (B) and all-cause mortality (C) in individuals with CKM syndrome stages 0–3.

Adjusted age, sex, education level, occupation, marital Status, sleep duration, smoke status, drink status, SBP, DBP, BMI, cancer, hypertension, dyslipidemia treatment.

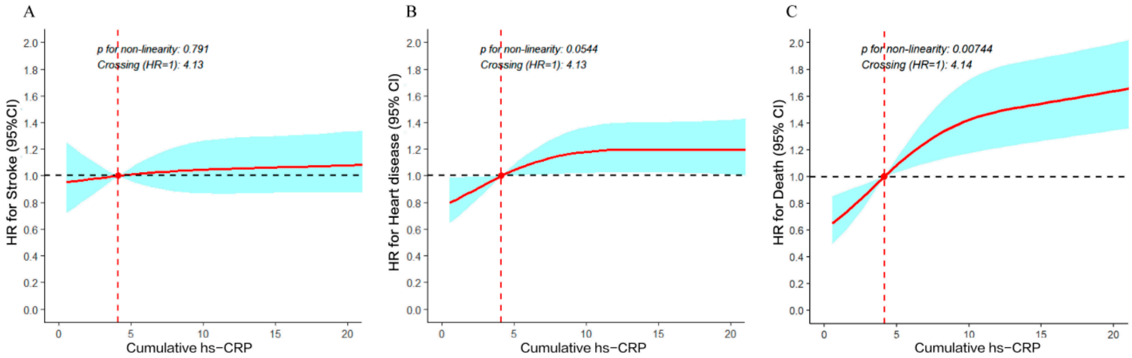

Figure S8 RCS analysis of the association of cumulative hs-CRP with stroke (A), heart disease (B) and all-cause mortality (C) in individuals with CKM syndrome stages 0–3.

Adjusted age, sex, education level, occupation, marital Status, sleep duration, smoke status, drink status, SBP, DBP, BMI, cancer, hypertension, dyslipidemia treatment.

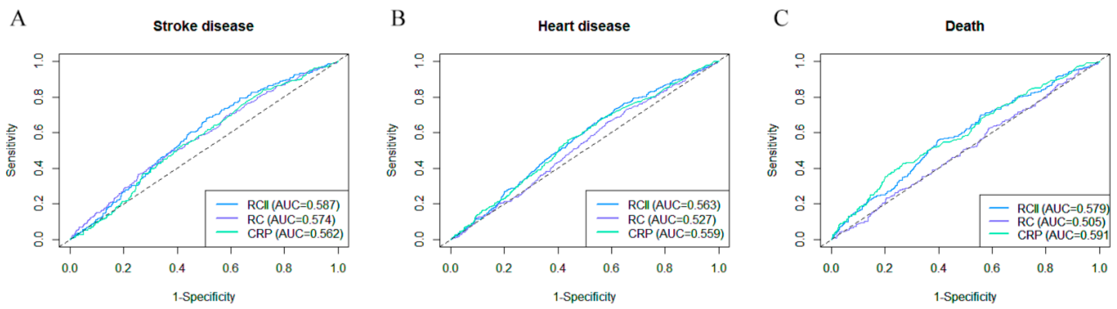

Figure S9 Receiver operating characteristic (ROC) curves comparing the discriminative performance of RCII, RC and hs-CRP for incident stroke (A), heart disease (B) and all-cause mortality (C) in individuals with CKM syndrome stages 0–3.

Adjusted age, sex, education level, occupation, marital Status, sleep duration, smoke status, drink status, SBP, DBP, BMI, cancer, hypertension, dyslipidemia treatment.

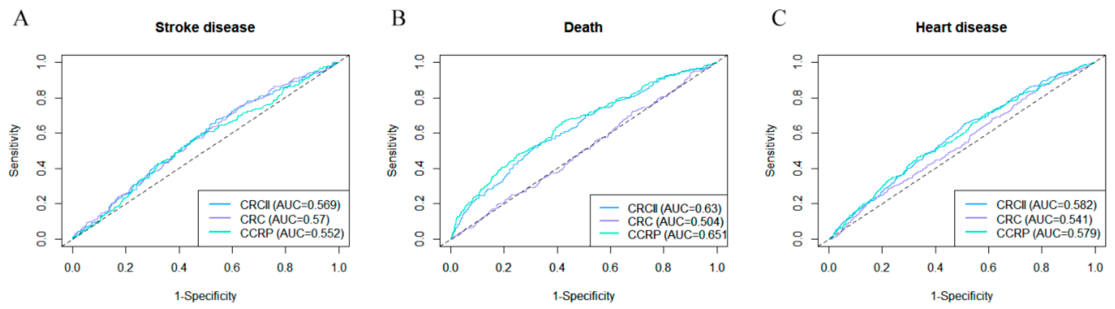

Figure S10 Receiver operating characteristic (ROC) curves comparing the discriminative performance of cumulative RCII, cumulative RC and cumulative hs-CRP for incident stroke (A), all-cause mortality (B) and heart disease (C) in individuals with CKM syndrome stages 0–3.

Adjusted age, sex, education level, occupation, marital Status, sleep duration, smoke status, drink status, SBP, DBP, BMI, cancer, hypertension, dyslipidemia treatment.

Table S1. Baseline characteristics of the study population with and without heart disease.

Characteristic	Total (N=6994)	No (N=6547)	Yes (N=447)	*P* value
**Age, mean (SD), years**	59.15 (9.32)	59.09 (9.38)	60.06 (8.49)	0.032
**Sex, N (%)**				<0.001
Female	3660 (52.3)	3384 (51.7)	276 (61.7)	
Male	3334 (47.7)	3163 (48.3)	171 (38.3)	
**Education level, N (%)**				0.132
Illiteracy	1996 (28.5)	1854 (28.3)	142 (31.8)	
Non-illiterate	4998 (71.5)	4693 (71.7)	305 (68.2)	
**Occupation, N (%)**				0.728
Famer	4382 (62.7)	4098 (62.6)	284 (63.5)	
Non-farmer	2612 (37.3)	2449 (37.4)	163 (36.5)	
**Marital Status, N (%)**				0.369
Married	6174 (88.3)	5773 (88.2)	401 (89.7)	
Others	820 (11.7)	774 (11.8)	46 (10.3)	
**Sleep duration, N (%), hours/day**				0.049
<7	3503 (50.1)	3254 (49.7)	249 (55.7)	
7-9	3186 (45.6)	3005 (45.9)	181 (40.5)	
≥9	305 (4.4)	288 (4.4)	17 (3.8)	
**Smoke status, N (%)**				0.085
Smoker	2781 (39.8)	2621 (40.0)	160 (35.8)	
Non-smoker	4213 (60.2)	3926 (60.0)	287 (64.2)	
**Drink status, N (%)**				0.066
Yes	5360 (76.6)	5001 (76.4)	359 (80.3)	

No	1634 (23.4)	1546 (23.6)	88 (19.7)	
**SBP, mean (SD), mmHg**	130.60 (21.42)	130.39 (21.38)	133.71 (21.78)	0.002
**DBP, mean (SD), mmHg**	75.91 (12.12)	75.82 (12.11)	77.31 (12.27)	0.012
**BMI, kg/m^2**	23.60 (10.54)	23.56 (10.84)	24.08 (4.22)	0.314
**Cancer, N (%)**				0.371
No	6927 (99.0)	6482 (99.0)	445 (99.6)	
Yes	67 (1.0)	65 (1.0)	2 (0.4)	
**Hypertension, N (%)**				<0.001
No	5554 (79.4)	5237 (80.0)	317 (70.9)	
Yes	1440 (20.6)	1310 (20.0)	130 (29.1)	
**Dyslipidemia treatment, N (%)**				0.389
No	6783 (97.0)	6353 (97.0)	430 (96.2)	
Yes	211 (3.0)	194 (3.0)	17 (3.8)	
**CKM, N (%)**				0.007
0	669 (9.6)	644 (9.8)	25 (5.6)	
1	1076 (15.4)	1014 (15.5)	62 (13.9)	
2	4663 (66.7)	4336 (66.2)	327 (73.2)	
3	586 (8.4)	553 (8.4)	33 (7.4)	
**RC, mean (SD), mg/dL**	25.94 (24.28)	25.93 (24.51)	26.05 (20.74)	0.916
**Hs-CRP, mean (SD), mg/L**	2.48 (5.71)	2.46 (5.71)	2.74 (5.81)	0.330
**RCII, mean (SD)**	6.20 (13.26)	6.18 (13.33)	6.47 (12.30)	0.657

Abbreviations: SD, standard deviation; BMI, body mass index; SBP, systolic blood pressure; DBP, diastolic blood pressure; CKM, cardiovascular–kidney–metabolic; RC, remnant cholesterol; hs-CRP, high-sensitivity C-reactive protein; RCII, remnant cholesterol inflammatory index.

Table S2. Baseline characteristics of the study individuals in death.

Characteristic	Total (N=7245)	Q1 (N=2415)	Q2 (N=2415)	Q3 (N=2415)	*P* value
**Age, mean (SD), years**	59.24 (9.31)	58.54 (9.25)	59.13 (9.29)	60.04 (9.34)	<0.001
**Sex, N (%)**					0.520
Female	3787 (52.3)	1269 (52.5)	1240 (51.3)	1278 (52.9)	
Male	3458 (47.7)	1146 (47.5)	1175 (48.7)	1137 (47.1)	
**Education level, N (%)**					0.521
Illiteracy	2060 (28.4)	697 (28.9)	666 (27.6)	697 (28.9)	
Non-illiterate	5185 (71.6)	1718 (71.1)	1749 (72.4)	1718 (71.1)	
**Occupation, N (%)**					<0.001
Famer	4506 (62.2)	1669 (69.1)	1503 (62.2)	1334 (55.2)	
Non-farmer	2739 (37.8)	746 (30.9)	912 (37.8)	1081 (44.8)	
**Marital Status, N (%)**					0.257
Married	6394 (88.3)	2136 (88.4)	2147 (88.9)	2111 (87.4)	
Others	851 (11.7)	279 (11.6)	268 (11.1)	304 (12.6)	
**Sleep duration, N (%), hours/day**					0.761
<7	3643 (50.3)	1194 (49.4)	1222 (50.6)	1227 (50.8)	
7-9	3292 (45.4)	1122 (46.5)	1084 (44.9)	1086 (45.0)	
≥9	310 (4.3)	99 (4.1)	109 (4.5)	102 (4.2)	
**Smoke status, N (%)**					0.008
Smoker	2877 (39.7)	898 (37.2)	986 (40.8)	993 (41.1)	
Non-smoker	4368 (60.3)	1517 (62.8)	1429 (59.2)	1422 (58.9)	
**Drink status, N (%)**					0.327
Yes	5565 (76.8)	1860 (77.0)	1831 (75.8)	1874 (77.6)	

No	1680 (23.2)	555 (23.0)	584 (24.2)	541 (22.4)	
**SBP, mean (SD), mmHg**	130.69 (21.46)	126.88 (20.56)	130.95 (21.26)	134.25 (21.90)	<0.001
**DBP, mean (SD), mmHg**	75.95 (12.11)	73.89 (11.99)	76.16 (12.05)	77.80 (11.97)	<0.001
**BMI, kg/m^2**	23.60 (10.38)	22.30 (3.24)	24.05 (17.11)	24.44 (4.22)	<0.001
**Cancer, N (%)**					0.380
No	7175 (99.0)	2397 (99.3)	2390 (99.0)	2388 (98.9)	
Yes	70 (1.0)	18 (0.7)	25 (1.0)	27 (1.1)	
**Hypertension, N (%)**					<0.001
No	5696 (78.6)	2067 (85.6)	1908 (79.0)	1721 (71.3)	
Yes	1549 (21.4)	348 (14.4)	507 (21.0)	694 (28.7)	
**Dyslipidemia treatment, N (%)**					<0.001
No	7003 (96.7)	2363 (97.8)	2348 (97.2)	2292 (94.9)	
Yes	242 (3.3)	52 (2.2)	67 (2.8)	123 (5.1)	
**CKM, N (%)**					<0.001
0	680 (9.4)	377 (15.6)	195 (8.1)	108 (4.5)	
1	1112 (15.3)	593 (24.6)	353 (14.6)	166 (6.9)	
2	4841 (66.8)	1282 (53.1)	1688 (69.9)	1871 (77.5)	
3	612 (8.4)	163 (6.7)	179 (7.4)	270 (11.2)	
**RC, mean (SD), mg/dL**	25.87 (23.86)	12.48 (8.28)	23.93 (13.43)	41.20 (32.27)	<0.001
**Hs-CRP, mean (SD), mg/L**	2.48 (5.70)	0.60 (0.51)	1.23 (0.99)	5.60 (9.02)	<0.001
**RCII, mean (SD)**	6.14 (12.82)	0.59 (0.30)	2.20 (0.74)	15.62 (18.87)	<0.001

Abbreviations: Q1, quantile1; Q2, quantile2; Q3, quantile3; SD, standard deviation; BMI, body mass index; SBP, systolic blood pressure; DBP, diastolic blood pressure; CKM, cardiovascular-kidney-metabolic; RC, remnant cholesterol; hs-CRP, high-sensitivity C-reactive protein; RCII, remnant cholesterol inflammatory index.

Table S3. Baseline characteristics of the study population with and without all-cause death.

Characteristic	Total (N=67245)	No (N=6592)	Yes (N=653)	*P* value
**Age, mean (SD), years**	59.24 (9.31)	58.33 (8.72)	68.38 (10.16)	<0.001
**Sex, N (%)**				
Female	3787 (52.3)	3540 (53.7)	247 (37.8)	<0.001
Male	3458 (47.7)	3052 (46.3)	406 (62.2)	
**Education level, N (%)**				
Illiteracy	2060 (28.4)	1783 (27.0)	277 (42.4)	<0.001
Non-illiterate	5185 (71.6)	4809 (73.0)	376 (57.6)	
**Occupation, N (%)**				
Famer	4506 (62.2)	4194 (63.6)	312 (47.8)	<0.001
Non-farmer	2739 (37.8)	2398 (36.4)	341 (52.2)	
**Marital Status, N (%)**				
Married	6394 (88.3)	5923 (89.9)	471 (72.1)	<0.001
Others	851 (11.7)	669 (10.1)	182 (27.9)	
**Sleep duration, N (%), hours/day**				
<7	3643 (50.3)	3298 (50.0)	345 (52.8)	0.302
7-9	3292 (45.4)	3014 (45.7)	278 (42.6)	
≥9	310 (4.3)	280 (4.2)	30 (4.6)	
**Smoke status, N (%)**				
Smoker	2877 (39.7)	2520 (38.2)	357 (54.7)	<0.001
Non-smoker	4368 (60.3)	4072 (61.8)	296 (45.3)	
**Drink status, N (%)**				
Yes	5565 (76.8)	5097 (77.3)	468 (71.7)	0.001

No	1680 (23.2)	1495 (22.7)	185 (28.3)	
**SBP, mean (SD), mmHg**	130.69 (21.46)	129.87 (20.93)	139.03 (24.68)	<0.001
**DBP, mean (SD), mmHg**	75.95 (12.11)	75.86 (11.99)	76.80 (13.25)	0.059
**BMI, kg/m^2**	23.60 (10.38)	23.77 (10.80)	21.87 (3.97)	<0.001
**Cancer, N (%)**				
No	7175 (99.0)	6535 (99.1)	640 (98.0)	0.009
Yes	70 (1.0)	57 (0.9)	13 (2.0)	
**Hypertension, N (%)**				
No	5696 (78.6)	5237 (79.4)	459 (70.3)	<0.001
Yes	1549 (21.4)	1355 (20.6)	194 (29.7)	
**Dyslipidemia treatment, N (%)**				
No	7003 (96.7)	6373 (96.7)	630 (96.5)	0.875
Yes	242 (3.3)	219 (3.3)	23 (3.5)	
**CKM, N (%)**				
0	680 (9.4)	612 (9.3)	68 (10.4)	<0.001
1	1112 (15.3)	1066 (16.2)	46 (7.0)	
2	4841 (66.8)	4384 (66.5)	457 (70.0)	
3	612 (8.4)	530 (8.0)	82 (12.6)	
**RC, mean (SD), mg/dL**	25.87 (23.86)	26.01 (24.00)	24.41 (22.41)	0.101
**Hs-CRP, mean (SD), mg/L**	2.48 (5.70)	2.27 (5.21)	4.57 (9.02)	<0.001
**RCII, mean (SD)**	6.14 (12.82)	5.80 (12.19)	9.59 (17.65)	<0.001

Abbreviations: SD, standard deviation; BMI, body mass index; SBP, systolic blood pressure; DBP, diastolic blood pressure; CKM, cardiovascular-kidney-metabolic; RC, remnant cholesterol; hs-CRP, high-sensitivity C-reactive protein; RCII, remnant cholesterol inflammatory index.

Table S4. Associations of RC and hs-CRP with the risk of stroke, heart disease and all-cause mortality.

Outcome	Events /Total	Model 1			Model 2			Model 3		
		HR (95%CI)	*P* value	PAF% (95% CI)	HR (95%CI)	*P* value	PAF% (95% CI)	HR (95%CI)	*P* value	PAF% (95% CI)
**RC**										
Stroke										
Q1	68/2423	Reference	-	-	Reference	-	-	Reference	-	-
Q2	91/2411	1.37 (0.99, 1.87)	0.051	8.8 (-0.03, 16.5)	1.36 (0.99, 1.87)	0.054	9.6 (-0.2, 16.2)	1.26 (0.92, 1.73)	0.151	6.7 (-2.6, 14.9)
Q3	118/2411	1.77 (1.32, 2.39)	<0.001	18.6 (9.5, 26.4)	1.82 (1.34, 2.45)	<0.001	19.5 (10.3, 27.3)	1.57 (1.16, 2.13)	0.003	15.0 (5.2, 23.3)
P trend			0.809			0.936			0.830	
Heart disease										
Q1	120/2353	Reference	-	-	Reference	-	-	Reference	-	-
Q2	171/2310	1.47 (1.16, 1.85)	0.002	12.2 (5.0, 18.6)	1.44 (1.14, 1.82)	0.002	11.8 (4.5, 18.3)	1.40 (1.11, 1.77)	0.005	11.0 (3.5, 17.7)
Q3	156/2331	1.33 (1.05, 1.68)	0.020	8.6 (1.4, 15.1)	1.29 (1.02, 1.64)	0.035	7.9 (0.6, 14.4)	1.21 (0.95, 1.54)	0.124	5.8 (-1.7, 12.6)
P trend			0.022			0.056			0.115	
All-cause mortality										
Q1	232/2423	Reference	-	-	Reference	-	-	Reference	-	-
Q2	226/2411	0.98 (0.81, 1.17)	0.810	-0.8 (-7.4, 5.4)	1.01 (0.84, 1.21)	0.936	0.2 (-6.1, 6.2)	1.02 (0.85, 1.23)	0.830	0.6 (-5.7, 6.5)
Q3	195/2411	0.85 (0.70, 1.02)	0.085	-5.4 (-11.9, 0.8)	0.97 (0.80, 1.17)	0.719	-1.1 (-7.6, 5.1)	1.01 (0.83, 1.24)	0.902	0.4 (-6.3, 6.8)
P trend			0.809			0.936			0.830	
**hs-CRP**										
Stroke										
Q1	76/2433	Reference	-	-	Reference	-	-	Reference	-	-
Q2	90/2401	1.23 (0.91, 1.68)	0.174	6.2 (-2.9, 14.1)	1.16 (0.85, 1.57)	0.352	4.4 (-5.1, 12.8)	1.09 (0.80, 1.48)	0.587	2.7 (-7.3, 11.6)
Q3	111/2411	1.55 (1.16, 2.08)	0.003	14.5 (5.2, 22.6)	1.40 (1.04, 1.88)	0.025	11.2 (1.5, 19.8)	1.23 (0.91, 1.66)	0.175	6.9 (-3.2, 15.8)
P trend			<0.001			0.028			0.005	

Heart disease										
Q1	123/2358	Reference	-	-	Reference	-	-	Reference	-	-
Q2	159/2317	1.36 (1.07, 1.72)	0.011	9.4 (23, 15.8)	1.32 (1.04, 1.67)	0.022	8.6 (1.3, 15.1)	1.28 (1.01, 1.62)	0.043	7.7 (0.2, 14.5)
Q3	165/2319	1.43 (1.13, 1.80)	0.003	11.2 (4.0, 17.6)	1.38 (1.09, 1.75)	0.007	10.2 (2.8, 16.9)	1.30 (1.02, 1.65)	0.033	8.3 (0.7, 15.1)
P trend			0.120			0.154			0.268	
All-cause mortality										
Q1	143/2433	Reference	-	-	Reference	-	-	Reference	-	-
Q2	203/2401	1.48 (1.19, 1.83)	<0.001	10.0 (4.7, 14.7)	1.27 (1.03, 1.58)	0.0281	6.9 (0.8, 12.4)	1.36 (1.10, 1.69)	0.005	8.7 (2.7, 14.1)
Q3	307/2411	2.27 (1.86, 2.76)	<0.001	26.9 (21.1, 31.4)	1.69 (1.38, 2.06)	<0.001	17.1 (10.9, 22.6)	1.78 (1.46, 2.18)	<0.001	18.6 (12.5, 23.9)
P trend			<0.001			0.028			0.005	

Abbreviations: RC, remnant cholesterol; hs-CRP, high-sensitivity C-reactive protein; PAF, population attributable fraction; HR, hazard ratio; CI, confidence interval; Q1, quantile1; Q2, quantile2; Q3, quantile3; SBP, systolic blood pressure; DBP, diastolic blood pressure; BMI, body mass index.

Model 1: Unadjusted.

Model 2: Adjusted age, sex, education level, occupation, marital Status, sleep duration, smoke status, drink status,

Model 3: Adjusted age, sex, education level, occupation, marital Status, sleep duration, smoke status, drink status, SBP, DBP, BMI, cancer, hypertension, dyslipidemia treatment.

Table S5. Associations of cumulative RC and cumulative CRP with the risk of stroke, heart disease and all-cause mortality.

Outcome	Events /Total	Model 1			Model 2			Model 3		
		HR (95%CI)	*P* value	PAF% (95% CI)	HR (95%CI)	*P* value	PAF% (95% CI)	HR (95%CI)	*P* value	PAF% (95% CI)
**Cumulative RC**										
Stroke										
Q1	47/1569	Reference	-	-	Reference	-	-	Reference	-	-
Q2	80/1569	1.73 (1.21, 2.48)	0.003	16.2 (6.0, 24.7)	1.70 (1.18, 2.45)	0.004	15.4 (5.3, 23,6)	1.62 (1.12, 2.33)	0.010	14.4 (3.8, 23.1)
Q3	81/1569	1.76 (1.23, 2.52)	0.002	16.8 (6.6, 25.3)	1.85 (1.28, 2.67)	0.001	18.7 (8.1, 27.3)	1.68 (1.16, 2.43)	0.006	15.7 (4.8, 24.8)
P trend			0.003			0.004			0.010	
Heart disease										
Q1	102/1569	Reference	-	-	Reference	-	-	Reference	-	-
Q2	117/1569	1.16 (0.89, 1.51)	0.273	4.6 (-3.9, 12.3)	1.11 (0.85, 1.45)	0.461	3.2 (-5.5, 11.0)	1.07 (0.82, 1.40)	0.614	2.2 (-6.7, 10.3)
Q3	128/1569	1.28 (0.98, 1.66)	0.065	8.0 (-0.6, 15.7)	1.23 (0.94, 1.61)	0.123	6.9 (-2.0, 14.9)	1.15 (0.88, 1.50)	0.320	4.5 (-4.6, 12.8)
P trend			0.273			0.461			0.614	
All-cause mortality										
Q1	71/1633	Reference	-	-	Reference	-	-	Reference	-	-
Q2	82/1633	1.16 (0.85, 1.60)	0.349	5.3 (-6.1 ,15.4)	1.25 (0.91, 1.73)	0.171	7.3 (-3.3, 16.4)	1.28 (0.93, 1.77)	0.135	7.9 (-2.7, 16.8)
Q3	65/1633	0.93 (0.66, 1.30)	0.651	-2.4 (-13.5, 7.6)	1.22 (0.86, 1.73)	0.257	6.5 (-4.8, 16.5)	1.27 (0.89, 1.82)	0.191	7.7 (-4.0, 17.9)
P trend			0.349			0.171			0.135	
**Cumulative hs-CRP**										
Stroke										
Q1	61/1572	Reference	-	-	Reference	-	-	Reference	-	-
Q2	68/1566	1.12 (0.79, 1.59)	0.511	3.6 (-7.5, 13.2)	1.08 (0.76, 1.53)	0.659	2.5 (-9.0, 12.5)	1.04 (0.74, 1.47)	0.820	1.3 (-10.5, 11.8)
Q3	79/1569	1.31 (0.94, 1.83)	0.112	9.1 (-2.2, 18.8)	1.21 (0.86, 1.69)	0.273	6.3 (-5.3, 16.4)	1.09 (0.78, 1.54)	0.607	3.0 (-8.8, 13.4)

P trend			0.511			0.659			0.820	
Heart disease										
Q1	95/1572	Reference	-	-	Reference	-	-	Reference	-	-
Q2	118/1566	1.26 (0.96, 1.64)	0.099	6.9 (-1.4, 14.3)	1.25 (0.95, 1.64)	0.109	6.8 (-1.6, 14.2)	1.21 (0.92, 1.59)	0.170	5.9 (-2.7, 13.6)
Q3	134/1569	1.44 (1.10, 1.87)	0.007	11.8 (3.3, 19.2)	1.42 (1.09, 1.85)	0.010	11.3 (2.8, 18.9)	1.32 (1.01, 1.73)	0.040	9.2 (0.4, 17.0)
P trend			0.099			0.108			0.170	
All-cause mortality										
Q1	45/1633	Reference	-	-	Reference	-	-	Reference	-	-
Q2	64/1634	1.42 (0.97, 2.08)	0.070	8.6 (-0.8, 16.4)	1.40 (0.95, 2.06)	0.085	9.1 (-1.6, 18.0)	1.42 (0.96, 2.08)	0.077	9.3 (-1.4, 18.4)
Q3	109/1632	2.48 (1.76, 3.52)	<0.001	30.1 (20.1, 38.0)	1.94 (1.37, 2.76)	<0.001	21.5 (10.8, 30.1)	1.94 (1.36, 2.77)	<0.001	21.5 (10.8, 30.1)
P trend			0.070			0.085			0.077	

Abbreviations: RC, remnant cholesterol; hs-CRP, high-sensitivity C-reactive protein; PAF, population attributable fraction; HR, hazard ratio; CI, confidence interval; Q1, quantile1; Q2, quantile2; Q3, quantile3; SBP, systolic blood pressure; DBP, diastolic blood pressure; BMI, body mass index.

Model 1: Unadjusted.

Model 2: Adjusted age, sex, education level, occupation, marital Status, sleep duration, smoke status, drink status,

Model 3: Adjusted age, sex, education level, occupation, marital Status, sleep duration, smoke status, drink status, SBP, DBP, BMI, cancer, hypertension, dyslipidemia treatment.

Table S6. Associations of K-means RC and K-means CRP with the risk of stroke, heart disease and all-cause mortality.

Outcome	Events /Total	Model 1			Model 2			Model 3		
		HR (95%CI)	*P* value	PAF% (95% CI)	HR (95%CI)	*P* value	PAF% (95% CI)	HR (95%CI)	*P* value	PAF% (95% CI)
**K-means RC**										
Stroke										
Cluster1	31/978	Reference	-	-	Reference	-	-	Reference	-	-
Cluster2	39/778	1.60 (1.00, 2.56)	0.051	7.0 (-0.0, 12.5)	1.73 (1.07, 2.79)	0.025	8.5 (1.2, 14.1)	1.52 (0.94, 2.47)	0.089	6.5 (-1.1, 12.4)
Cluster3	90/1742	1.66 (1.10, 2.50)	0.015	17.3 (3.9, 26.9)	1.68 (1.11, 2.53)	0.014	17.5 (4.1, 26.9)	1.54 (1.02, 2.33)	0.039	15.1 (0.8, 25.3)
Cluster4	48/1209	1.26 (0.80, 1.98)	0.319	4.7 (-5.2, 12.2)	1.22 (0.78, 1.93)	0.383	4.0 (-5.7, 11.3)	1.16 (0.74, 1.83)	0.516	3.1 (-7.2, 10.9)
P trend			0.051			0.025			0.089	
Heart disease										
Cluster1	65/978	Reference	-	-	Reference	-	-	Reference	-	-
Cluster2	67/778	1.31 (0.93, 1.85)	0.116	4.6 (-1.3, 9.6)	1.28 (0.91, 1.81)	0.163	4.2 (-1.8, 9.2)	1.17 (0.82, 1.66)	0.381	2.6 (-3.5, 7.9)
Cluster3	138/1742	1.21 (0.90, 1.62)	0.209	6.9 (-4.3, 15.8)	1.18 (0.88, 1.59)	0.275	6.0 (-5.3, 15.1)	1.12 (0.83, 1.51)	0.447	4.3 (-7.5, 13.8)
Cluster4	77/1209	0.96 (0.69, 1.33)	0.792	-1.0 (-9.2, 5.8)	0.96 (0.69, 1.34)	0.833	-0.8 (-9.2, 6.1)	0.94 (0.67, 1.31)	0.714	-1.5 (-10.1, 5.8)
P trend			0.116			0.163			0.381	
All-cause mortality										
Cluster1	35/826	0.94 (0.60, 1.46)	0.776	-1.0 (-9.0, 5.7)	1.35 (0.86, 2.12)	0.199	5.2 (-2.8, 11.9)	1.37 (0.86, 2.19)	0.188	5.6 (-2.8, 12.7)
Cluster2	58/1254	1.03 (0.70, 1.51)	0.896	0.6 (-10.6, 9.6)	1.05 (0.71, 1.55)	0.807	1.1 (-8.9, 8,8)	1.02 (0.69, 1.51)	0.930	0.4 (-9.6, 8.1)
Cluster3	46/1014	Reference	-	-	Reference	-	-	Reference	-	-
Cluster4	79/1805	0.97 (0.68, 1.40)	0.891	-0.9(-16.1, 10.9)	1.18 (0.82, 1.72)	0.375	5.9 (-8.1, 16.5)	1.19 (0.82, 1.74)	0.360	6.2 (-8.0, 16.9)
P trend			0.896			0.807			0.930	
**K-means hs-CRP**										
Stroke										
Cluster1	20/484	1.10 (0.68, 1.80)	0.695	0.9 (-3.5, 5.5)	1.04 (0.64, 1.71)	0.872	0.4 (-4.0, 5.0)	0.92 (0.56, 1.52)	0.755	-0.7 (-5.0, 3.9)

Cluster2	79/2092	Reference	-	-	Reference	-	-	Reference	-	-
Cluster3	86/1726	1.33 (0.98, 1.80)	0.068	10.2 (-0.8, 19.6)	1.29 (0.95, 1.75)	0.108	9.2 (-2.2, 19.0)	1.21 (0.89, 1.65)	0.219	7.2 (-4.5, 17.4)
Cluster4	23/405	1.51 (0.95, 2.41)	0.080	3.8 (-0.4, 8.1)	1.33 (0.83, 2.12)	0.234	2.5 (-1.6, 6.7)	1.21 (0.76, 1.93)	0.430	1.7 (-2.4, 5.9)
P trend			0.695			0.872			0.755	
Heart disease										
Cluster1	44/484	1.44 (1.03, 2.03)	0.035	3.9 (0.3, 7.6)	1.44 (1.02, 2.03)	0.036	4.0 (0.2, 7.7)	1.35 (0.96, 1.91)	0.087	3.3 (-0.5, 7.0)
Cluster2	135/2092	Reference	-	-	Reference	-	-	Reference	-	-
Cluster3	126/1726	1.14 (0.89, 1.45)	0.306	4.3 (-4.1, 11.9)	1.12 (0.88, 1.43)	0.373	3.8 (-4.7, 11.4)	1.07 (0.84, 1.37)	0.580	2.4 (-6.3, 10.2)
Cluster4	42/405	1.63 (1.15, 2.31)	0.006	4.7 (1.3, 8.1)	1.62 (1.14, 2.30)	0.007	4.7 (1.3, 8.1)	1.51 (1.06, 2.15)	0.022	4.0 (0.6, 7.4)
P trend			0.035			0.036			0.087	
All-cause mortality										
Cluster1	71/1798	1.33 (0.95, 1.87)	0.095	8.0 (-1.5, 16.0)	1.28 (0.91, 1.80)	0.160	7.4 (-3.3, 16.5)	1.28 (0.91, 1.81)	0.156	7.5 (-3.3, 16.8)
Cluster2	29/444	2.21 (1.43, 3.43)	<0.001	7.2 (3.2, 11.9)	1.54 (0.99, 2.40)	0.056	3.6 (-0.1, 7.1)	1.55 (0.99, 2.41)	0.053	2.7 (-0.1, 7.3)
Cluster3	54/498	3.86 (2.69, 5.54)	<0.001	18.9 (14.2, 22.8)	3.03 (2.10, 4.38)	<0.001	15.0 (10.1, 19.2)	3.00 (2.08, 4.35)	<0.001	14.7 (9.9, 19.0)
Cluster4	64/2159	Reference	-	-	Reference	-	-	Reference	-	-
P trend			0.095			0.160			0.156	

Abbreviations: RC, remnant cholesterol; hs-CRP, high-sensitivity C-reactive protein; PAF, population attributable fraction; HR, hazard ratio; CI, confidence interval; SBP, systolic blood pressure; DBP, diastolic blood pressure; BMI, body mass index.

Model 1: Unadjusted.

Model 2: Adjusted age, sex, education level, occupation, marital Status, sleep duration, smoke status, drink status,

Model 3: Adjusted age, sex, education level, occupation, marital Status, sleep duration, smoke status, drink status, SBP, DBP, BMI, cancer, hypertension, dyslipidemia treatment.

Table S7. Subgroup Analysis of RCII with the risk of stroke, heart disease and all-cause mortality.

	Q1	Q2		Q3		*P* value
		HR (95%CI)	*P* value	HR (95%CI)	*P* value	
**Stroke**						
Age						0.032
<60	Reference	1.48 (0.89, 2.44)	0.130	2.09 (1.30, 3.38)	0.003	
≥60	Reference	1.35 (0.89, 2.04)	0.160	1.17 (0.77, 1.77)	0.469	
Gender						0.713
Male	Reference	1.29 (0.82, 2.03)	0.275	1.58 (1.02, 2.46)	0.042	
Female	Reference	1.53 (0.97, 2.39)	0.065	1.52 (0.98, 2.37)	0.064	
EDU						0.338
Non-illiterate	Reference	1.40 (0.95, 2.08)	0.092	1.73 (1.18, 2.52)	0.005	
Illiterate	Reference	1.48 (0.86, 2.55)	0.158	1.25 (0.72, 2.18)	0.421	
Farming						0.151
Yes	Reference	1.14 (0.76, 1.70)	0.521	1.56 (1.06, 2.28)	0.023	
No	Reference	1.97 (1.13, 3.42)	0.016	1.64 (0.94, 2.85)	0.079	
Marital status						0.401
Married	Reference	1.50 (1.06, 2.13)	0.023	1.53 (1.08, 2.17)	0.016	
Others	Reference	0.94 (0.42, 2.13)	0.884	1.63 (0.78, 3.38)	0.195	
Sleep time						0.909
<7h	Reference	1.31 (0.84, 2.06)	0.230	1.62 (1.06, 2.50)	0.027	
7-9h	Reference	1.53 (0.94, 2.50)	0.089	1.50 (0.92, 2.45)	0.104	
>9h	Reference	1.46 (0.38, 5.66)	0.584	0.96 (0.24, 3.88)	0.956	
Smoke						0.141
Yes	Reference	1.11 (0.68, 1.82)	0.668	1.68 (1.07, 2.64)	0.026	
No	Reference	1.67 (1.09, 2.56)	0.018	1.45 (0.94, 2.24)	0.089	

Drinking						0.686
Yes	Reference	1.31 (0.91, 1.90)	0.147	1.53 (1.07, 2.18)	0.021	
No	Reference	1.58 (0.82, 3.03)	0.173	1.50 (0.77, 2.90)	0.233	
CKM						0.841
0	Reference	0.48 (0.09, 2.48)	0.383	1.12 (0.22, 5.64)	0.891	
1	Reference	1.19 (0.50, 2.82)	0.695	1.60 (0.60, 4.26)	0.351	
2	Reference	1.57 (1.06, 2.31)	0.024	1.67 (1.14, 2.45)	0.008	
3	Reference	1.14 (0.37, 3.52)	0.815	1.12 (0.38, 3.28)	0.839	
**Heart disease**						
Age						0.301
<60	Reference	1.39 (0.99, 1.95)	0.054	1.58 (1.13, 2.22)	0.008	
≥60	Reference	1.35 (0.95, 1.91)	0.090	1.25 (0.88, 1.77)	0.206	
Gender						0.419
Male	Reference	1.68 (1.14, 2.49)	0.009	1.55 (1.03, 2.31)	0.035	
Female	Reference	1.20 (0.88, 1.63)	0.258	1.31 (0.97, 1.78)	0.078	
EDU						0.968
Non-illiterate	Reference	1.35 (1.00, 1.80)	0.047	1.42 (1.06, 1.90)	0.019	
Illiterate	Reference	1.38 (0.90, 2.12)	0.138	1.33 (0.87, 2.04)	0.192	
Farming						0.234
Yes	Reference	1.24 (0.92, 1.67)	0.153	1.47 (1.10, 1.97)	0.010	
No	Reference	1.58 (1.04, 2.41)	0.034	1.34 (0.88, 2.06)	0.174	
Marital status						0.599
Married	Reference	1.37 (1.06, 1.76)	0.015	1.35 (1.05, 1.75)	0.020	
Others	Reference	1.33 (0.59, 2.97)	0.490	1.86 (0.87, 3.98)	0.110	
Sleep time						0.571
<7h	Reference	1.23 (0.89, 1.70)	0.214	1.37 (0.99, 1.88)	0.054	

7-9h	Reference	1.51 (1.03, 2.22)	0.034	1.53 (1.04, 2.25)	0.032	
>9h	Reference	2.07 (0.61, 7.02)	0.246	0.91 (0.22, 3.71)	0.892	
Smoke						0.488
Yes	Reference	1.62 (1.06, 2.49)	0.027	1.81 (1.18, 2.78)	0.006	
No	Reference	1.24 (0.92, 1.67)	0.154	1.22 (0.91, 1.65)	0.182	
Drinking						0.102
Yes	Reference	1.55 (1.17, 2.04)	0.002	1.60 (1.21, 2.10)	<0.001	
No	Reference	0.87 (0.52, 1.46)	0.596	0.86 (0.51, 1.48)	0.594	
CKM						0.948
0	Reference	0.98 (0.38, 2.56)	0.970	1.37 (0.50, 3.76)	0.547	
1	Reference	1.49 (0.85, 2.61)	0.160	1.56 (0.76, 3.18)	0.223	
2	Reference	1.27 (0.95, 1.71)	0.107	1.30 (0.98, 1.74)	0.072	
3	Reference	1.55 (0.51, 4.69)	0.438	1.57 (0.53, 4.68)	0.417	
**All-cause mortality**						
Age						0.950
<60	Reference	1.29 (0.84, 1.98)	0.249	1.55 (1.00, 2.40)	0.050	
≥60	Reference	1.28 (1.01, 1.62)	0.040	1.67 (1.33, 2.08)	<0.001	
Gender						0.423
Male	Reference	1.45 (1.12, 1.88)	0.005	1.80 (1.40, 2.32)	<0.001	
Female	Reference	1.03 (0.73, 1.44)	0.878	1.43 (1.04, 1.98)	0.028	
EDU						0.484
Non-illiterate	Reference	1.41 (1.08, 1.85)	0.013	1.68 (1.29, 2.19)	<0.001	
Illiterate	Reference	1.12 (0.81, 1.53)	0.493	1.62 (1.20, 2.18)	0.002	
Farming						0.128
Yes	Reference	1.24 (0.94, 1.64)	0.122	1.37 (1.03, 1.81)	0.030	

No	Reference	1.35 (0.99, 1.85)	0.055	2.02 (1.51, 2.70)	<0.001	
Marital status						0.751
Married	Reference	1.34 (1.05, 1.71)	0.018	1.78 (1.40, 2.25)	<0.001	
Others	Reference	1.11 (0.75, 1.64)	0.594	1.36 (0.94, 1.97)	0.100	
Sleep time						0.662
<7h	Reference	1.25 (0.94, 1.66)	0.122	1.71 (1.31, 2.24)	<0.001	
7-9h	Reference	1.41 (1.03, 1.93)	0.033	1.57 (1.15, 2.15)	0.005	
>9h	Reference	0.71 (0.24, 2.11)	0.537	1.59 (0.60, 4.19)	0.349	
Smoke						0.678
Yes	Reference	1.40 (1.06, 1.85)	0.019	1.70 (1.30, 2.23)	<0.001	
No	Reference	1.16 (0.85, 1.57)	0.348	1.57 (1.17, 2.10)	0.003	
Drinking						0.217
Yes	Reference	1.19 (0.94, 1.51)	0.157	1.50 (1.19, 1.89)	<0.001	
No	Reference	1.59 (1.06, 2.37)	0.023	2.17 (1.48, 3.18)	<0.001	
CKM						0.004
0	Reference	1.15 (0.60, 2.23)	0.670	3.60 (2.02, 6.42)	<0.001	
1	Reference	1.46 (0.70, 3.05)	0.311	3.70 (1.82, 7.53)	<0.001	
2	Reference	1.25 (0.99, 1.59)	0.065	1.31 (1.03, 1.67)	0.027	
3	Reference	1.05 (0.46-2.42)	0.906	1.94 (0.93, 4.06)	0.079	

Abbreviations: RCII, remnant cholesterol inflammatory index; HR, hazard ratio; CI, confidence interval; Q1, quantile1; Q2, quantile2; Q3, quantile3.

Table S8. Multivariable Cox regression analysis of RCII, RC and hs-CRP association with the risk of stroke, heart disease and all-cause mortality in individuals with CKM syndrome (stages 0–3): after excluding individuals with missing covariates.

Outcome	Events /Total	Model 1		Model 2		Model 3	
		HR (95%CI)	*P* value	HR (95%CI)	*P* value	HR (95%CI)	*P* value
**RCII**							
Stroke							
Q1	49/1690	Reference	-	Reference	-	Reference	-
Q2	67/1689	1.41 (0.97, 2.03)	0.069	1.36 (0.94, 1.97)	0.103	1.26 (0.87, 1.83)	0.217
Q3	78/1690	1.65 (1.16, 2.36)	0.006	1.56 (1.09, 2.24)	0.015	1.33 (0.92, 1.91)	0.131
Heart disease							
Q1	84/1690	Reference	-	Reference	-	Reference	-
Q2	117/1689	1.44 (1.09, 1.91)	0.011	1.41 (1.06, 1.87)	0.017	1.36 (1.03, 1.81)	0.032
Q3	132/1690	1.64 (1.25, 2.16)	<0.001	1.57 (1.19, 2.08)	0.001	1.45 (1.10, 1.92)	0.010
All-cause mortality							
Q1	102/1757	Reference	-	Reference	-	Reference	-
Q2	147/1756	1.09 (0.85, 1.41)	0.491	1.1 (0.85, 1.42)	0.483	1.08 (0.84, 1.41)	0.542
Q3	188/1756	1.09 (0.86, 1.39)	0.467	1.11 (0.87, 1.42)	0.410	1.15 (0.89, 1.47)	0.283
**RC**							
Stroke							
Q1	50/1699	Reference	-	Reference	-	Reference	-
Q2	67/1685	1.35 (0.94, 1.95)	0.107	1.34 (0.93, 1.93)	0.122	1.22 (0.84, 1.76)	0.296
Q3	77/1685	1.57 (1.10, 2.25)	0.013	1.58 (1.11, 2.27)	0.0118	1.37 (0.96, 1.97)	0.0859
Heart disease							
Q1	94/1699	Reference	-	Reference	-	Reference	-
Q2	117/1685	1.26 (0.96, 1.65)	0.097	1.25 (0.95, 1.64)	0.1113	1.20 (0.92, 1.58)	0.184

Q3	122/1685	1.33 (1.02, 1.74)	0.037	1.3 (0.99, 1.70)	0.058	1.21 (0.92, 1.59)	0.1697
All-cause mortality							
Q1	140/1766	Reference	-	Reference	-	Reference	-
Q2	163/1754	0.93 (0.74, 1.16)	0.509	0.93 (0.74, 1.17)	0.5378	0.93 (0.74, 1.18)	0.5494
Q3	134/1749	0.92 (0.72, 1.17)	0.498	0.92 (0.71, 1.17)	0.4905	0.93 (0.72, 1.21)	0.5948
**hs-CRP**							
Stroke							
Q1	56/1723	Reference	-	Reference	-	Reference	-
Q2	65/1662	1.23 (0.86, 1.76)	0.255	1.17 (0.81, 1.67)	0.401	1.10 (0.76, 1.57)	0.621
Q3	73/1684	1.39 (0.98, 1.96)	0.066	1.26 (0.88, 1.79)	0.202	1.11 (0.77, 1.58)	0.578
Heart disease							
Q1	88/1723	Reference	-	Reference	-	Reference	-
Q2	126/1662	1.54 (1.17, 2.02)	0.002	1.49 (1.13, 1.96)	0.0043	1.44 (1.10, 1.90)	0.0091
Q3	119/1684	1.44 (1.10, 1.90)	0.009	1.38 (1.05, 1.83)	0.0224	1.29 (0.97, 1.71)	0.0767
All-cause mortality							
Q1	88/1784	Reference	-	Reference	-	Reference	-
Q2	141/1738	0.92 (0.71, 1.20)	0.553	0.89 (0.68, 1.17)	0.4093	0.91 (0.69, 1.21)	0.52
Q3	208/1747	0.94 (0.73, 1.20)	0.612	0.94 (0.73, 1.22)	0.6329	0.96 (0.74, 1.25)	0.7603

Abbreviations: RCII, remnant cholesterol inflammatory index; hs-CRP, high-sensitivity C-reactive protein; RC, remnant cholesterol; PAF, population attributable fraction; HR, hazard ratio; CI, confidence interval; Q1, quantile1; Q2, quantile2; Q3, quantile3; SBP, systolic blood pressure; DBP, diastolic blood pressure; BMI, body mass index.

Model 1: Unadjusted.

Model 2: Adjusted age, sex, education level, occupation, marital Status, sleep duration, smoke status, drink status,

Model 3: Adjusted age, sex, education level, occupation, marital Status, sleep duration, smoke status, drink status, SBP, DBP, BMI, cancer, hypertension, dyslipidemia treatment.

Table S9. Multivariable Cox regression analysis of RCII, RC and hs-CRP association with the risk of stroke, heart disease and all-cause mortality in individuals with CKM syndrome (stages 0–3): after excluding participants who was observed the outcomes within 2 years of baseline.

Outcome	Events /Total	Model 1		Model 2		Model 3	
		HR (95%CI)	*P* value	HR (95%CI)	*P* value	HR (95%CI)	*P* value
**RCII**							
Stroke							
Q1	50/2269	Reference	-	Reference	-	Reference	-
Q2	82/2268	1.68 (1.18, 2.39)	0.004	1.62 (1.14, 2.31)	0.007	1.50 (1.05, 2.14)	0.025
Q3	88/2268	1.84 (1.30, 2.60)	<0.001	1.74 (1.22, 2.46)	0.002	1.49 (1.04, 2.12)	0.028
Heart disease							
Q1	88/2269	Reference	-	Reference	-	Reference	-
Q2	123/2268	1.44 (1.09, 1.89)	0.009	1.43 (1.08, 1.88)	0.011	1.37 (1.04, 1.81)	0.024
Q3	143/2268	1.71 (1.31, 2.23)	<0.001	1.68 (1.28, 2.19)	<0.001	1.56 (1.19, 2.05)	0.001
All-cause mortality							
Q1	151/2379	Reference	-	Reference	-	Reference	-
Q2	190/2378	1.29 (1.04, 1.6)	0.019	1.25 (1.01, 1.55)	0.041	1.30 (1.05, 1.61)	0.018
Q3	233/2378	1.59 (1.29, 1.95)	<0.001	1.44 (1.17, 1.77)	<0.001	1.54 (1.25, 1.90)	<0.001
**RC**							
Stroke							
Q1	52/2297	Reference	-	Reference	-	Reference	-
Q2	75/2245	1.49 (1.04, 2.12)	0.028	1.48 (1.04, 2.11)	0.030	1.38 (0.97, 1.96)	0.078
Q3	93/2263	1.83 (1.31, 2.58)	<0.001	1.87 (1.33, 2.63)	<0.001	1.65 (1.17, 2.33)	0.004
Heart disease							
Q1	96/2297	Reference	-	Reference	-	Reference	-

Q2	136/2245	1.47 (1.13, 1.90)	0.004	1.46 (1.12, 1.89)	0.005	1.41 (1.09, 1.84)	0.010
Q3	122/2263	1.30 (1.00, 1.70)	0.053	1.29 (0.98, 1.69)	0.066	1.20 (0.92, 1.58)	0.180
All-cause mortality							
Q1	200/2382	Reference	-	Reference	-	Reference	-
Q2	204/2380	1.02 (0.84, 1.24)	0.813	1.05 (0.87, 1.28)	0.596	1.07 (0.88, 1.30)	0.522
Q3	170/2373	0.86 (0.70, 1.05)	0.135	0.97 (0.79, 1.2)	0.793	1.02 (0.82, 1.26)	0.851
**hs-CRP**							
Stroke							
Q1	65/2310	Reference	-	Reference	-	Reference	-
Q2	70/2231	1.14 (0.81, 1.59)	0.456	1.06 (0.75, 1.49)	0.747	1.00 (0.71, 1.41)	0.980
Q3	85/2264	1.40 (1.01, 1.93)	0.043	1.25 (0.9, 1.73)	0.181	1.11 (0.8, 1.54)	0.534
Heart disease							
Q1	94/2310	Reference	-	Reference	-	Reference	-
Q2	126/2231	1.43 (1.10, 1.87)	0.008	1.4 (1.07, 1.83)	0.015	1.35 (1.03, 1.77)	0.028
Q3	134/2264	1.53 (1.18, 2.00)	0.002	1.49 (1.14, 1.95)	0.003	1.41 (1.08, 1.85)	0.012
All-cause mortality							
Q1	134/2416	Reference	-	Reference	-	Reference	-
Q2	181/2341	1.42 (1.14, 1.78)	0.002	1.22 (0.98, 1.53)	0.077	1.31 (1.04, 1.64)	0.020
Q3	259/2378	2.03 (1.65, 2.50)	<0.001	1.52 (1.24, 1.88)	<0.001	1.61 (1.30, 1.99)	<0.001

Abbreviations: RCII, remnant cholesterol inflammatory index; hs-CRP, high-sensitivity C-reactive protein; RC, remnant cholesterol; PAF, population attributable fraction; HR, hazard ratio; CI, confidence interval; Q1, quantile1; Q2, quantile2; Q3, quantile3; SBP, systolic blood pressure; DBP, diastolic blood pressure; BMI, body mass index.

Model 1: Unadjusted.

Model 2: Adjusted age, sex, education level, occupation, marital Status, sleep duration, smoke status, drink status,

Model 3: Adjusted age, sex, education level, occupation, marital Status, sleep duration, smoke status, drink status, SBP, DBP, BMI, cancer, hypertension,

dyslipidemia treatment.

Table S10. Associations of RCII, RC and hs-CRP with the risk of stroke, heart disease and all-cause mortality by using age as the time scale.

Outcome	Events /Total	Model 1		Model 2		Model 3	
		HR (95%CI)	*P* value	HR (95%CI)	*P* value	HR (95%CI)	*P* value
**RCII**							
Stroke							
Q1	63/2415	Reference	-	Reference	-	Reference	-
Q2	97/2415	1.54 (1.12, 2.12)	0.007	1.52 (1.11, 2.09)	0.010	1.39 (1.01, 1.91)	0.044
Q3	117/2415	1.84 (1.35, 2.50)	<0.001	1.8 (1.32, 2.46)	<0.001	1.53 (1.12, 2.10)	0.007
Heart disease							
Q1	115/2332	Reference	-	Reference	-	Reference	-
Q2	160/2331	1.43 (1.12, 1.82)	0.004	1.42 (1.12, 1.81)	0.004	1.37 (1.07, 1.74)	0.011
Q3	172/2331	1.53 (1.21, 1.94)	<0.001	1.5 (1.18, 1.91)	<0.001	1.4 (1.1, 1.78)	0.007
All-cause mortality							
Q1	166/2415	Reference	-	Reference	-	Reference	-
Q2	210/2415	1.23 (1.00, 1.51)	0.049	1.23 (1.00, 1.51)	0.045	1.29 (1.05, 1.58)	0.017
Q3	277/2415	1.56 (1.29, 1.90)	<0.001	1.55 (1.27, 1.88)	<0.001	1.66 (1.36, 2.02)	<0.001
**RC**							
Stroke							
Q1	68/2423	Reference	-	Reference	-	Reference	-
Q2	91/2411	1.35 (0.99, 1.85)	0.062	1.34 (0.98, 1.84)	0.069	1.25 (0.91, 1.71)	0.171
Q3	118/2411	1.80 (1.33, 2.42)	<0.001	1.78 (1.32, 2.41)	<0.001	1.56 (1.15, 2.11)	0.004
Heart disease							
Q1	120/2353	Reference	-	Reference	-	Reference	-
Q2	171/2310	1.46 (1.15, 1.84)	0.002	1.43 (1.13, 1.81)	0.003	1.39 (1.1, 1.76)	0.006

Q3	156/2331	1.33 (1.05, 1.69)	0.018	1.28 (1.01, 1.63)	0.044	1.2 (0.94, 1.53)	0.142
All-cause mortality							
Q1	232/2423	Reference	-	Reference	-	Reference	-
Q2	226/2411	0.98 (0.81, 1.17)	0.804	0.99 (0.82, 1.19)	0.917	1.01 (0.84, 1.21)	0.930
Q3	195/2411	0.94 (0.78, 1.14)	0.557	0.97 (0.80, 1.17)	0.736	1.02 (0.83, 1.24)	0.880
**hs-CRP**							
Stroke							
Q1	76/2433	Reference	-	Reference	-	Reference	-
Q2	90/2401	1.15 (0.85, 1.56)	0.375	1.14 (0.84, 1.55)	0.407	1.08 (0.79, 1.46)	0.642
Q3	111/2411	1.42 (1.06, 1.90)	0.020	1.39 (1.03, 1.86)	0.031	1.22 (0.91, 1.65)	0.187
Heart disease							
Q1	123/2358	Reference	-	Reference	-	Reference	-
Q2	159/2317	1.32 (1.04, 1.67)	0.023	1.31 (1.03, 1.66)	0.026	1.27 (1.00, 1.61)	0.049
Q3	165/2319	1.38 (1.09, 1.74)	0.007	1.38 (1.09, 1.75)	0.007	1.30 (1.03, 1.65)	0.030
All-cause mortality							
Q1	143/2433	Reference	-	Reference	-	Reference	-
Q2	203/2401	1.25 (1.01, 1.55)	0.042	1.25 (1.01, 1.55)	0.043	1.35 (1.09, 1.68)	0.007
Q3	307/2411	1.72 (1.41, 2.11)	<0.001	1.66 (1.36, 2.03)	<0.001	1.76 (1.44, 2.16)	<0.001

Abbreviations: RCII, remnant cholesterol inflammatory index; hs-CRP, high-sensitivity C-reactive protein; RC, remnant cholesterol; PAF, population attributable fraction; HR, hazard ratio; CI, confidence interval; Q1, quantile1; Q2, quantile2; Q3, quantile3; SBP, systolic blood pressure; DBP, diastolic blood pressure; BMI, body mass index.

Model 1: Unadjusted.

Model 2: Adjusted age, sex, education level, occupation, marital Status, sleep duration, smoke status, drink status,

Model 3: Adjusted age, sex, education level, occupation, marital Status, sleep duration, smoke status, drink status, SBP, DBP, BMI, cancer, hypertension, dyslipidemia treatment.

Table S11. Multivariable Cox regression analysis of RCII, RC and hs-CRP association with the risk of stroke, heart disease and all-cause mortality in individuals with CKM syndrome (stages 0–3): using dichotomized exposure variable

Outcome	Events /Total	Model 1		Model 2		Model 3	
		HR (95%CI)	*P* value	HR (95%CI)	*P* value	HR (95%CI)	*P* value
**RCII**							
Stroke							
Low	108/3497	Reference	-	Reference	-	Reference	-
High	169/3497	1.61 (1.26, 2.05)	<0.001	1.54 (1.21, 1.96)	<0.001	1.35 (1.06, 1.73)	0.016
Heart disease							
Low	191/3497	Reference	-	Reference	-	Reference	-
High	256/3497	1.38 (1.14, 1.66)	<0.001	1.34 (1.11, 1.62)	0.003	1.26 (1.04, 1.53)	0.017
All-cause mortality							
Low	263/3623	Reference	-	Reference	-	Reference	-
High	390/3622	1.52 (1.30, 1.78)	<0.001	1.37 (1.17, 1.60)	<0.001	1.44 (1.23, 1.69)	<0.001
**RC**							
Stroke							
Low	116/3502	Reference	-	Reference	-	Reference	-
High	161/3492	1.40 (1.10, 1.78)	0.006	1.43 (1.12, 1.81)	0.004	1.30 (1.02, 1.66)	0.032
Heart disease							
Low	205/3502	Reference	-	Reference	-	Reference	-
High	242/3492	1.19 (0.99, 1.43)	0.066	1.17 (0.97, 1.41)	0.108	1.12 (0.93, 1.35)	0.248
All-cause mortality							
Low	350/3638	Reference	-	Reference	-	Reference	-
High	303/3607	0.87 (0.75, 1.02)	0.078	0.98 (0.84, 1.14)	0.766	1.02 (0.87, 1.20)	0.795

**hs-CRP**							
Stroke							
Low	116/3501	Reference	-	Reference	-	Reference	-
High	161/3493	1.43 (1.13, 1.82)	0.003	1.34 (1.05, 1.70)	0.017	1.21 (0.95, 1.54)	0.121
Heart disease							
Low	186/3501	Reference	-	Reference	-	Reference	-
High	261/3493	1.45 (1.20, 1.75)	<0.001	1.43 (1.18, 1.72)	<0.001	1.36 (1.12, 1.65)	0.002
All-cause mortality							
Low	251/3623	Reference	-	Reference	-	Reference	-
High	402/3622	1.65 (1.41, 1.93)	<0.001	1.36 (1.16, 1.60)	<0.001	1.41 (1.20, 1.65)	<0.001

Abbreviations: RCII, remnant cholesterol inflammatory index; hs-CRP, high-sensitivity C-reactive protein; RC, remnant cholesterol; PAF, population attributable fraction; HR, hazard ratio; CI, confidence interval; Q1, quantile1; Q2, quantile2; Q3, quantile3; SBP, systolic blood pressure; DBP, diastolic blood pressure; BMI, body mass index.

Model 1: Unadjusted.

Model 2: Adjusted age, sex, education level, occupation, marital Status, sleep duration, smoke status, drink status,

Model 3: Adjusted age, sex, education level, occupation, marital Status, sleep duration, smoke status, drink status, SBP, DBP, BMI, cancer, hypertension, dyslipidemia treatment.

Table S12. Multivariable Cox regression analysis of RCII, RC and hs-CRP association with the risk of stroke, heart disease and all-cause mortality in individuals with CKM syndrome (stages 0–3): using quartiles exposure variable

Outcome	Events /Total	Model 1		Model 2		Model 3	
		HR (95%CI)	*P* value	HR (95%CI)	*P* value	HR (95%CI)	*P* value
**RCII**							
Stroke							
Q1	44/1750	Reference	-	Reference	-	Reference	-
Q2	64/1747	1.49 (1.01, 2.18)	0.043	1.44 (0.98, 2.12)	0.061	1.36 (0.92, 1.99)	0.122
Q3	84/1748	1.96 (1.36, 2.82)	<0.001	1.86 (1.29, 2.68)	<0.001	1.63 (1.13, 2.35)	0.010
Q4	85/1749	2.03 (1.41, 2.92)	<0.001	1.91 (1.32, 2.75)	<0.001	1.58 (1.09, 2.29)	0.015
Heart disease							
Q1	87/1750	Reference	-	Reference	-	Reference	-
Q2	104/1747	1.22 (0.92, 1.63)	0.166	1.22 (0.92, 1.62)	0.171	1.19 (0.89, 1.58)	0.232
Q3	128/1748	1.52 (1.15, 1.99)	0.003	1.48 (1.13, 1.95)	0.005	1.40 (1.06, 1.85)	0.017
Q4	128/1749	1.55 (1.18, 2.03)	0.002	1.49 (1.13, 1.96)	0.004	1.37 (1.04, 1.81)	0.026
All-cause mortality							
Q1	119/1813	Reference	-	Reference	-	Reference	-
Q2	144/1810	1.24 (0.97, 1.58)	0.083	1.22 (0.95, 1.55)	0.113	1.25 (0.98, 1.59)	0.078
Q3	176/1811	1.52 (1.20, 1.91)	<0.001	1.37 (1.08, 1.73)	0.009	1.45 (1.14, 1.83)	0.002
Q4	214/1811	1.89 (1.51, 2.36)	<0.001	1.67 (1.34, 2.09)	<0.001	1.81 (1.44, 2.28)	<0.001
**RC**							
Stroke							
Q1	45/1753	Reference	-	Reference	-	Reference	-
Q2	71/1749	1.60 (1.10, 2.32)	0.014	1.58 (1.09, 2.3)	0.016	1.48 (1.02, 2.15)	0.041
Q3	71/1751	1.59 (1.10, 2.31)	0.015	1.6 (1.10, 2.32)	0.015	1.45 (1.00, 2.12)	0.051

Q4	90/1741	2.04 (1.43, 2.92)	<0.001	2.1 (1.47, 3.01)	<0.001	1.81 (1.26, 2.60)	0.001
Heart disease							
Q1	92/1753	Reference	-	Reference	-	Reference	-
Q2	113/1749	1.25 (0.95, 1.64)	0.114	1.23 (0.93, 1.62)	0.145	1.19 (0.9, 1.57)	0.218
Q3	132/1751	1.46 (1.12, 1.90)	0.006	1.41 (1.08, 1.84)	0.012	1.35 (1.03, 1.77)	0.027
Q4	110/1741	1.22 (0.92, 1.61)	0.163	1.19 (0.90, 1.57)	0.229	1.10 (0.83, 1.46)	0.505
All-cause mortality							
Q1	178/1821	Reference	-	Reference	-	Reference	-
Q2	172/1817	0.97 (0.79, 1.20)	0.808	1.00 (0.81, 1.24)	0.978	1.02 (0.83, 1.26)	0.833
Q3	155/1801	0.88 (0.71, 1.09)	0.242	0.96 (0.77, 1.19)	0.683	1.00 (0.80, 1.24)	0.975
Q4	148/1806	0.84 (0.68, 1.05)	0.118	1.00 (0.80, 1.25)	0.979	1.08 (0.86, 1.35)	0.516
**hs-CRP**							
Stroke							
Q1	51/1797	Reference	-	Reference	-	Reference	-
Q2	65/1704	1.38 (0.95, 1.99)	0.087	1.30 (0.90, 1.88)	0.164	1.27 (0.88, 1.83)	0.204
Q3	87/1744	1.82 (1.29, 2.57)	<0.001	1.70 (1.20, 2.40)	0.003	1.55 (1.09, 2.19)	0.015
Q4	74/1749	1.56 (1.09, 2.23)	0.014	1.39 (0.97, 1.99)	0.076	1.22 (0.85, 1.75)	0.293
Heart disease							
Q1	98/1797	Reference	-	Reference	-	Reference	-
Q2	88/1704	0.96 (0.72, 1.29)	0.804	0.93 (0.7, 1.25)	0.644	0.92 (0.69, 1.23)	0.569
Q3	140/1744	1.53 (1.18, 1.98)	0.001	1.48 (1.14, 1.92)	0.003	1.42 (1.09, 1.84)	0.009
Q4	121/1749	1.33 (1.02, 1.73)	0.037	1.27 (0.97, 1.67)	0.079	1.20 (0.91, 1.57)	0.198
All-cause mortality							
Q1	108/1852	Reference	-	Reference	-	Reference	-
Q2	143/1771	1.42 (1.11, 1.82)	0.006	1.19 (0.93, 1.53)	0.168	1.25 (0.97, 1.61)	0.084

Q3	146/1818	1.42 (1.10, 1.82)	0.006	1.17 (0.91, 1.50)	0.216	1.22 (0.95, 1.58)	0.116
Q4	256/1804	2.57 (2.05, 3.22)	<0.001	1.80 (1.43, 2.25)	<0.001	1.91 (1.52, 2.40)	<0.001

Abbreviations: RCII, remnant cholesterol inflammatory index; hs-CRP, high-sensitivity C-reactive protein; RC, remnant cholesterol; PAF, population attributable fraction; HR, hazard ratio; CI, confidence interval; Q1, quantile1; Q2, quantile2; Q3, quantile3; SBP, systolic blood pressure; DBP, diastolic blood pressure; BMI, body mass index.

Model 1: Unadjusted.

Model 2: Adjusted age, sex, education level, occupation, marital Status, sleep duration, smoke status, drink status,

Model 3: Adjusted age, sex, education level, occupation, marital Status, sleep duration, smoke status, drink status, SBP, DBP, BMI, cancer, hypertension, dyslipidemia treatment.

Table S13. Multivariable Cox regression analysis of RCII, RC and hs-CRP association with the risk of stroke, heart disease and all-cause mortality in individuals with CKM syndrome (stages 0–3): using quintiles exposure variable

Outcome	Events /Total	Model 1		Model 2		Model 3	
		HR (95%CI)	*P* value	HR (95%CI)	*P* value	HR (95%CI)	*P* value
**RCII**							
Stroke							
Q1	34/1399	Reference	-	Reference	-	Reference	-
Q2	46/1399	1.36 (0.87, 2.12)	0.173	1.32 (0.85, 2.06)	0.218	1.25 (0.80, 1.94)	0.331
Q3	60/1398	1.82 (1.20, 2.77)	0.005	1.74 (1.14, 2.65)	0.010	1.53 (1.00, 2.34)	0.048
Q4	71/1399	2.14 (1.42, 3.22)	<0.001	2.05 (1.36, 3.08)	<0.001	1.76 (1.16, 2.66)	0.007
Q5	66/1399	2.04 (1.35, 3.09)	<0.001	1.89 (1.24, 2.86)	0.003	1.53 (1.00, 2.33)	0.048
Heart disease							
Q1	67/1399	Reference	-	Reference	-	Reference	-
Q2	76/1399	1.14 (0.82, 1.58)	0.443	1.12 (0.81, 1.56)	0.489	1.09 (0.79, 1.52)	0.594
Q3	93/1398	1.44 (1.05, 1.97)	0.023	1.41 (1.03, 1.93)	0.034	1.33 (0.97, 1.82)	0.080
Q4	112/1399	1.72 (1.27, 2.32)	<0.001	1.67 (1.24, 2.27)	<0.001	1.57 (1.15, 2.13)	0.004
Q5	99/1399	1.55 (1.14, 2.12)	0.005	1.49 (1.09, 2.03)	0.013	1.35 (0.98, 1.85)	0.064
All-cause mortality							
Q1	101/1449	Reference	-	Reference	-	Reference	-
Q2	95/1449	0.94 (0.71, 1.25)	0.683	0.95 (0.72, 1.26)	0.712	0.96 (0.73, 1.28)	0.801
Q3	131/1449	1.34 (1.03, 1.74)	0.027	1.28 (0.99, 1.66)	0.064	1.34 (1.03, 1.74)	0.031
Q4	135/1449	1.36 (1.05, 1.76)	0.020	1.28 (0.99, 1.66)	0.062	1.35 (1.04, 1.76)	0.023
Q5	191/1449	1.99 (1.57, 2.54)	<0.001	1.71 (1.34, 2.18)	<0.001	1.88 (1.47, 2.41)	<0.001
**RC**							
Stroke							

Q1	36/1409	Reference	-	Reference	-	Reference	-
Q2	49/1396	1.38 (0.90, 2.13)	0.140	1.37 (0.89, 2.11)	0.150	1.28 (0.83, 1.97)	0.265
Q3	54/1401	1.51 (0.99, 2.31)	0.054	1.5 (0.98, 2.29)	0.060	1.37 (0.89, 2.09)	0.149
Q4	66/1390	1.88 (1.25, 2.82)	0.002	1.9 (1.27, 2.86)	0.002	1.69 (1.12, 2.55)	0.012
Q5	72/1398	2.04 (1.37, 3.05)	<0.001	2.12 (1.42, 3.17)	<0.001	1.78 (1.18, 2.67)	0.006
Heart disease							
Q1	78/1409	Reference	-	Reference	-	Reference	-
Q2	73/1396	0.95 (0.69, 1.31)	0.754	0.93 (0.68, 1.28)	0.670	0.9 (0.66, 1.24)	0.532
Q3	107/1401	1.39 (1.04, 1.87)	0.026	1.36 (1.02, 1.82)	0.039	1.3 (0.97, 1.75)	0.077
Q4	95/1390	1.24 (0.92, 1.68)	0.153	1.20 (0.88, 1.62)	0.245	1.13 (0.83, 1.53)	0.436
Q5	94/1398	1.23 (0.91, 1.66)	0.179	1.20 (0.89, 1.62)	0.239	1.1 (0.81, 1.5)	0.535
All-cause mortality							
Q1	140/1457	Reference	-	Reference	-	Reference	-
Q2	137/1445	0.99 (0.78, 1.25)	0.934	1.03 (0.81, 1.31)	0.797	1.07 (0.84, 1.36)	0.580
Q3	142/1452	1.02 (0.81, 1.28)	0.889	1.07 (0.85, 1.36)	0.550	1.11 (0.88, 1.41)	0.380
Q4	113/1448	0.81 (0.63, 1.04)	0.096	0.9 (0.7, 1.15)	0.398	0.96 (0.75, 1.24)	0.781
Q5	121/1443	0.87 (0.69, 1.12)	0.280	1.1 (0.86, 1.41)	0.451	1.17 (0.91, 1.51)	0.221
**hs-CRP**							
Stroke							
Q1	40/1414	Reference	-	Reference	-	Reference	-
Q2	57/1404	1.48 (0.99, 2.21)	0.059	1.42 (0.94, 2.12)	0.093	1.36 (0.91, 2.04)	0.137
Q3	48/1381	1.25 (0.82, 1.91)	0.290	1.17 (0.77, 1.78)	0.472	1.10 (0.72, 1.68)	0.660
Q4	75/1403	1.97 (1.34, 2.89)	<0.001	1.82 (1.23, 2.67)	0.003	1.62 (1.10, 2.38)	0.015
Q5	57/1392	1.53 (1.02, 2.29)	0.040	1.34 (0.89, 2.02)	0.157	1.15 (0.76, 1.74)	0.503
Heart disease							

Q1	84/1414	Reference	-	Reference	-	Reference	-
Q2	63/1404	0.77 (0.55, 1.06)	0.111	0.75 (0.54, 1.04)	0.080	0.73 (0.53, 1.01)	0.060
Q3	103/1381	1.29 (0.97, 1.72)	0.084	1.23 (0.92, 1.64)	0.169	1.18 (0.88, 1.58)	0.256
Q4	98/1403	1.22 (0.91, 1.63)	0.190	1.18 (0.88, 1.59)	0.258	1.12 (0.83, 1.50)	0.462
Q5	99/1392	1.26 (0.94, 1.69)	0.117	1.20 (0.89, 1.61)	0.228	1.11 (0.83, 1.50)	0.485
All-cause mortality							
Q1	82/1458	Reference	-	Reference	-	Reference	-
Q2	103/1448	1.30 (0.97, 1.73)	0.079	1.14 (0.86, 1.53)	0.364	1.16 (0.87, 1.55)	0.316
Q3	114/1450	1.43 (1.08, 1.90)	0.014	1.19 (0.89, 1.58)	0.236	1.26 (0.95, 1.68)	0.114
Q4	141/1440	1.81 (1.38, 2.37)	<0.001	1.43 (1.09, 1.88)	0.011	1.52 (1.16, 2.01)	0.003
Q5	213/1449	2.78 (2.15, 3.58)	<0.001	1.92 (1.49, 2.49)	<0.001	2.00 (1.54, 2.59)	<0.001

Abbreviations: RCII, remnant cholesterol inflammatory index; hs-CRP, high-sensitivity C-reactive protein; RC, remnant cholesterol; PAF, population attributable fraction; HR, hazard ratio; CI, confidence interval; Q1, quantile1; Q2, quantile2; Q3, quantile3; SBP, systolic blood pressure; DBP, diastolic blood pressure; BMI, body mass index.

Model 1: Unadjusted.

Model 2: Adjusted age, sex, education level, occupation, marital Status, sleep duration, smoke status, drink status,

Model 3: Adjusted age, sex, education level, occupation, marital Status, sleep duration, smoke status, drink status, SBP, DBP, BMI, cancer, hypertension, dyslipidemia treatment.
